# Sinomenine attenuates uremia vascular calcification by miR-143-5p

**DOI:** 10.1038/s41598-025-86055-2

**Published:** 2025-01-13

**Authors:** Fengyi Yu, Zhong Peng, Ning Gao, Zixu Tang, Zihao Liao, Song Zhao, Shuzhu Zhong, Gloria Umwiza, Hong Huang, Wei Long, Zhangxiu He

**Affiliations:** 1 Department of Nephrology, Yiyang Central Hospital, 118 Kangfubei Road, Yiyang, 413000 Hunan China; 2 Department of Gastroenterology, Yiyang Central Hospital, Yiyang, Hunan China; 3 Hengyang Medical School, University of South China, Hengyang, Hunan China

**Keywords:** Sinomenine, Vascular calcification, Uremia, RNA-seq, microRNAs, Medical research, Nephrology, Pathogenesis

## Abstract

Vascular calcification is considered to be a killer of the cardiovascular system, involved inflammation and immunity. There is no approved therapeutic strategy for the prevention of vascular calcification. Sinomenine exhibited anti-inflammatory and immunosuppressive effects. Objective of this study was to investigate the effect of sinomenine in vascular calcification and its potential molecular mechanism. Adenine-induced uremic rats were constructed and administrated with sinomenine. Optical clearing of aortas, alizarin red staining, von Kossa staining, calcification quantification, micro-CT analyses of vascular calcification were performed to analyze calcification in aortas. Administration of 40 mg/kg/d sinomenine effectively alleviated vascular calcification in uremic rats. The miRNA sequencing revealed differentially expressed miRNAs in aortas and bioinformatic analysis assisted with miRNA screening. We screened 9 differential expressed miRNAs and their predicted target genes. By qRT-PCR, we validated that the expression of rno-miR-143-5p was corresponding to our prediction. Sinomenine inhibited vascular smooth muscle cells (VSMCs) calcification, accompanied with miR-143-5p upregulation. MiR-143-5p mimic decreased VSMCs calcification in high phosphate condition. On the contrary, miR-143-5p inhibitor increased VSMCs calcification in high phosphate condition, which was inhibited by sinomenine. In chronic kidney disease patients with vascular calcification, the expression level of circulating miR-143-5p was lower than those without vascular calcification. Sinomenine significantly inhibited vascular calcification in VSMCs and uremic rat. MiR-143-5p was one of the collection of miRNAs modified by sinomenine in vascular calcification. Reduction of miR-143-5p in VSMCs was not only a concomitant phenomenon in pro-calcification condition but also contribute to VSMCs calcification. Circulating miR-143-5p was supposed to be a potential biomarker for vascular calcification in chronic kidney disease patients. In conclusion, sinomenine effectively alleviated vascular calcification, which was attributed to miR-143-5p regulation partly.

## Introduction

Vascular calcification, crystallization of hydroxyapatite deposited in the medial or intimal arterial wall, was thought to be a passive process in the past^1^. However, recent researches reveal that vascular calcification is an active process involved inflammation, osteogenic transformations and other factors^2^. Moreover, vascular calcification is considered to be a killer of the cardiovascular system, especially in chronic kidney disease patients^2,3^. Even today, the mechanisms of vascular calcification are still not fully understood and there is no approved therapeutic strategy for the prevention of vascular calcification. Thus, it is worth to explore applicable drugs for the treatment of vascular calcification and elucidate its related mechanism.

Sinomenine (CAS 115-53-5) is a pure alkaloid extracted from the roots and stems of Chinese herb Qingfengteng (scientific name: *Sinomenium acutum* (Thunb.) Rehd. et Wils.). Currently, sinomenine has been used in clinic in the forms of tablets or injection, such as Zhengqing Fengtongning^4^. Sinomenine exhibited anti-inflammatory and immunosuppressive functions^5^. Importantly, sinomenine regulated vascular smooth muscle cells (VSMCs) phenotypic switching including inhibition of VSMC migration, dedifferentiation and proliferation, which played a critical role in vascular calcification^6^. Nevertheless, there is little study about the effect of sinomenine on uremia vascular calcification.

Sinomenine regulated miR-324-5p and inhibited the suppression of invasion and metastasis in breast cancer cells^7^. Similarly, sinomenine decreased the level of miR-21 to inhibit lung cancer cell invasion^8^. MiRNAs involved messenger RNAs (mRNAs) degradation and translation repression and were significant regulators in diverse biological pathways^9^. Thus, regulated microRNAs (miRNAs) by sinomenine potentially played an important role in treatment. Chao et al. has screened differentially expressed miRNAs from aortas between uremic rats and healthy rats^10^. However, there is rare miRNA-sequence (miRNA-seq) information about vascular calcification with treatment of sinomenine.

The objective of this study was to explore the potential effect of sinomenine on uremia vascular calcification. We demonstrated that sinomenine alleviated vascular calcification in vivo and in vitro. Sinomenine administration led to miRNAs expression alteration, such as miR-143-5p. MiR-143-5p mimic significantly inhibited VSMC calcification. Our findings shed light on that sinomenine alleviated vascular calcification through miRNAs regulation, which also provided a prospective method for early diagnosis and treatment on vascular calcification.

## Materials and methods

### Reagent

For rat gavage, sinomenine hydrochloride (purity > 99%) was purchased from Zhengqing Pharmaceutical Group (Hunan, China) and dissolved in saline. For cell culture, sinomenine hydrochloride (purity > 99%) was purchased from APExBIO Technology Limited Liability Company (Houston, USA) and dissolved in double distilled water. Sodium thiosulfate (purity > 99%) was purchased from Sigma-Aldrich, Inc (Missouri, America) and dissolved in double distilled water.

### Animal models

All animal studies were performed according to ARRIVE (animal research: reporting in vivo experiments) guidelines (https://arriveguidelines.org). They were performed in accordance with protocols and regulations approved by the Ethics Committee of Yiyang Central Hospital and University of South China. Approval Number is 2021SK51811. An adenine-induced chronic kidney disease animal model was constructed. The rats were observed for 7 days at the start of the gavage administration. Eight-week-old male specific pathogen free Sprague-Dawley rats were obtained from the Experimental Animal Center of University of South China (Hengyang, China) and then randomly divided into 4 groups. Each group contained 7 rats. Adenine (Sigma, USA) was dissolved in distilled water to prepare the adenine suspension. The rats in the uremic chronic kidney disease group (CKD) were fed with a 1.8% high-phosphorus diet and given 2.5% adenine suspension (220 mg/kg/d) by gavage daily for 8 weeks. The rats were added 20 mg/kg/d sinomenine (Sin 20 mg) and 40 mg/kg/d sinomenine (Sin) daily from 5 week to 8 week additionally. The rats in the control group (Con) were fed with standard chow and given the same volume of saline gavage administration. Eight weeks later, they were sacrificed in an induction box for anesthesia. The induction box was provided with fresh air firstly. Subsequently, carbon dioxide displacement method was used gradually to induce anesthesia and euthanasia. Blood samples were collected and aortas were harvested. Blood urea nitrogen, creatinine, calcium, and phosphate were detected by an autoanalyzer (Hitachi, Japan) and the results were in Supplementary Figure S1. The connective and adipose tissues of aortas were removed, and the residual blood was immediately washed with pre-cooled normal saline. The aortas were collected for further vascular calcification analysis and miRNA-seq.

### Optical clearing of aortas

To evaluate vascular calcification optically, the harvested aortas were fixed in neutral buffered formalin for 3 days. Aortas were then immersed in SCALEVIEW-S (FUJIFILM, Japan) for 7 days to render translucent. Subsequently, the specimens were cut open with pins setting to retain their shapes. Aortas were stained in 2% alizarin red staining (Sigma, USA) for 30 min. Then the samples were washed in distilled water three times, and immersed in SCALEVIEW-S for further days. Finally, macroscopic images of specimens were recorded.

### Cell culture and in vitro calcification model

A7R5 cells were obtained from Cell Bank/Stem Cell Bank, Chinese Academy of Sciences (Shanghai, China). MOVAS-1 cells and T/G HA-VSMCs were obtained from ATCC (USA). A7R5 cells and MOVAS-1 cells were cultured in the Dulbecco’s modified Eagle’s medium (Gibco, USA) supplemented with 10% FBS (Gibco, USA), 100 U/mL penicillin, and 100 mg/mL streptomycin (Thermo Fisher, USA) at 37 °C in a humidified atmosphere containing 5% CO_2_. T/G HA-VSMCs were cultured in smooth muscle cell medium (ScienCell, USA) which contained basic medium, 2% fetal bovine serum, 1% smooth muscle cell growth supplement and 1% penicillin/streptomycin solution at 37 °C in a humidified atmosphere containing 5% CO_2_.

To establish the in vitro vascular calcification model, VSMCs were cultured in 6-well plates and treated with calcifying medium (3 mM Pi) for 7 days. The culture medium was renewed every 2 days.

### Cell counting kit-8 (CCK-8) assay for cell growth

A7R5 cells and MOVAS-1 cells were resuspended and incubated in a 96-well plate with 5 × 10^4^ cells per well, incubated with different concentration sinomenine from 24 h to 96 h at 37 °C in a humidified atmosphere containing 5% CO_2_. Subsequently, the cells were cultured for additional 1 h in fresh complete medium with CCK-8 reagent (Beyotime, China). Finally, cell proliferation was evaluated by optical density at 450 nm. All experiments were conducted in triplicate.

### Alizarin red staining

For alizarin red staining, aorta tissue was fixed in neutral buffered formalin firstly and then embedded in paraffin and 2–5 μm serial sections were cut using a microtome. Sections were stained with 2% alizarin red staining (Sigma, USA) for 30 min and washed with 0.2% acetic acid. For alizarin red staining, VSMCs in 6-well plates were fixed in 4% formaldehyde for 10 min at room temperature, exposed to 2% alizarin red staining for 30 min and washed with 0.2% acetic acid. Calcification deposition showed a reddish or purple color.

### Von kossa staining

For von Kossa staining, aorta tissue sections were incubated with 5% silver nitrate solution for 30 min, exposed to bright light for 15 min, washed and treated with 5% sodium thiosulfate.

### Calcification quantification

Aorta tissue and VSMCs lysis solution was obtained according to calcium colorimetric assay kit (Beyotime, China). The protein concentrations of aortas lysis and cell lysis were determined by a BCA protein assay kit (Thermo Fisher Scientific; No. 23225). To determine calcium accumulation of sample, aorta tissue and VSMCs lysis segments were subjected to colorimetric analysis according to manufacture introduction. The calcium content in lysis was normalized to µg Ca/mg protein weight.

### Alkaline phosphatase (ALP) activity

Cell lysis wad obtained according to Alkaline Phosphatase Assay kit (Beyotime, China). The supernatant was assayed for protein content and ALP activity. Enzyme activity was determined by measuring at 405 nm. Total ALP activity was normalized as U/mg protein.

### The micro-CT analysis of vascular calcification

The procedure of micro-CT analysis of vascular calcification was described previously^11^. When fixed in neutral buffered formalin for 24 h, aortas were examined with a desktop X-ray µCT scanner. Two-dimensional image projections were obtained and three-dimensional visualizations of vascular calcification were constructed. According to images projections, vascular calcifications were quantified.

### The miRNA sequencing and bioinformatic analysis

Total RNA was extracted from aortas of control group, uremic chronic kidney disease group and 40 mg/kg/day sinomenine group (Con, CKD and Sin). Each group processed three samples. RNA was used for the small RNA library construction, reverse transcribed to cDNA and performed polymerase chain reaction amplification. The polymerase chain reaction products ranging from 140 to 160 bp were isolated and purified as small RNA libraries, which was assessed on the Agilent Bioanalyzer 2100 system using DNA High Sensitivity Chips. The libraries were finally sequenced using the Illumina HiSeq X Ten platform.

When comparing the differentially expressed miRNAs profiles among three groups, fold change and *P*-values were calculated and used to identify significant differentially expressed miRNAs. Hierarchical clustering was utilized to display the differentially expressed miRNAs. The overlaps of miRNAs down-regulated in CKD compared with Con and miRNAs up-regulated in Sin compared with CKD were predicted to alleviated vascular calcification. The overlaps of miRNAs of up-regulated in CKD compared with Con and miRNAs down-regulated in Sin compared with CKD were predicted to promote vascular calcification. Venn diagram showed the overlap of differentially expressed miRNAs. MiRNA target prediction was performed by using three databases: miRDB, miRWalk, and miRBase. To improve prediction accuracy, the overlapped predicted target genes from the three databases were considered to represent the final results. To reveal correlations between miRNAs and predicted target genes in three aorta groups, miRNA-mRNA networks were constructed using the Cytoscape software manual (http://www.cytoscape.org). The GO and KEGG pathway analyses were performed based on miRNA target prediction by OE Biotech Co., Ltd. (Shanghai, China). Our raw data can be found on the BioProject (accession number: PRJNA977694).

### Cell transfection

Mimics and inhibitors of rno-miR-143-5p, mmu-miR-143-5p and cel-miR-67-3p (RiboBio, China) were transfected into cells using Lipofectamine 3000 (Invitrogen, USA) according to the manufacturer’s protocol. Transfection was conducted for 24 h and validated by qRT-PCR. All experiments were conducted in triplicate.

### Quantitative reverse transcription polymerase chain reaction (qRT-PCR)

Total RNA was extracted using TRIzol reagent (Invitrogen, USA) for cell and aorta tissue or using Plasma Circulating RNA Extraction Kit (Solarbio, China) for patient plasma following the manufacturer’s protocols. RNA was reverse-transcribed into cDNA using Mir-X™ miRNA First Strand Synthesis Kit (Takara, Japan) according to the manufacturer’s protocols. Subsequently, cDNA was quantified by the Mir-X™ miRNA qRT-PCR SYBR^®^Kit (Takara, Japan). U6 expression served as an internal control for miRNA expression for cell and aorta tissue. MiR-486-5p was abundant in chronic kidney disease patients and showed a stable expression as extensively^12^. Therefore, hsa-miR-486-5p was used for normalization of miRNA expression in patient plasma. We used the 2^− DDCt^ method to calculate the fold changes in expression. Primers for miRNAs are list in (Supplementary Table 1).

### Assembly of two clinical validation cohorts of chronic kidney disease patients with vascular calcification or without vascular calcification

Patients with chronic kidney disease were recruited if their estimated glomerular filtration < 60 ml/min/1.73 m^2^ that remained stable for at least 3 months, from Yiyang Central Hospital, between 20 March 2024 and 15 September 2024. Exclusion criteria for the cohort patients consisted of those received a renal transplant or peritoneal dialysis. Written informed consent was obtained from all of the participants. The current study was approved by the Yiyang Central Hospital, and the protocol adhered to the Declaration of Helsinki (2023XYLH085).

We recorded clinical data from enrolled participants comprised demographic profile (age, gender and hemodialysis duration), comorbidities (diabetes mellitus, hypertension, heart failure, coronary artery disease, peripheral vascular disease and old cerebrovascular event), physical parameter (body mass index, systolic blood pressure and diastolic blood pressure), medication (aspirin, clopidogrel, statin, calcium-based phosphate binders and active vitamin D), and laboratory parameters (hemoglobin, albumin, calcium, phosphate, Ca-P product, intact parathyroid hormone (iPTH), total cholesterol, triglyceride, high-density lipoprotein and low density lipoprotein). For patients receiving hemodialysis, we collected their plasma and laboratory parameters detected before hemodialysis.

For vascular calcification phenotype determination in patients, we gauged participants’ aortic calcification as described^10^.

### Statistical analysis

Shapiro-Wilk test was used to examine whether the variables conformed to normal distribution. Normally distributed continuous variables are presented as means ± standard deviations. For non-normally distributed variables, we presented data in median with 25% percentile and 75% percentile. Student’s t-test was used for normally distributed continuous variables to analyzed between-group differences. One-way analysis of variance was used to compare data of > 2 groups if variables were normally distributed. For comparing non-normally distributed variables of 2 or > 2 groups, we used Mann-Whitney U test or Kruskal-Wallis test. In mixed analysis of variance, we used Sidak’s multiple comparisons test. All statistical analyses were performed using SPSS version 18.0, GraphPad Prism 8 and R software, with a 2-sided *P*-value < 0.05 being considered significant.

## Results

### Sinomenine alleviated vascular calcification in adenine-induced uremic rats

**Fig. 1:**
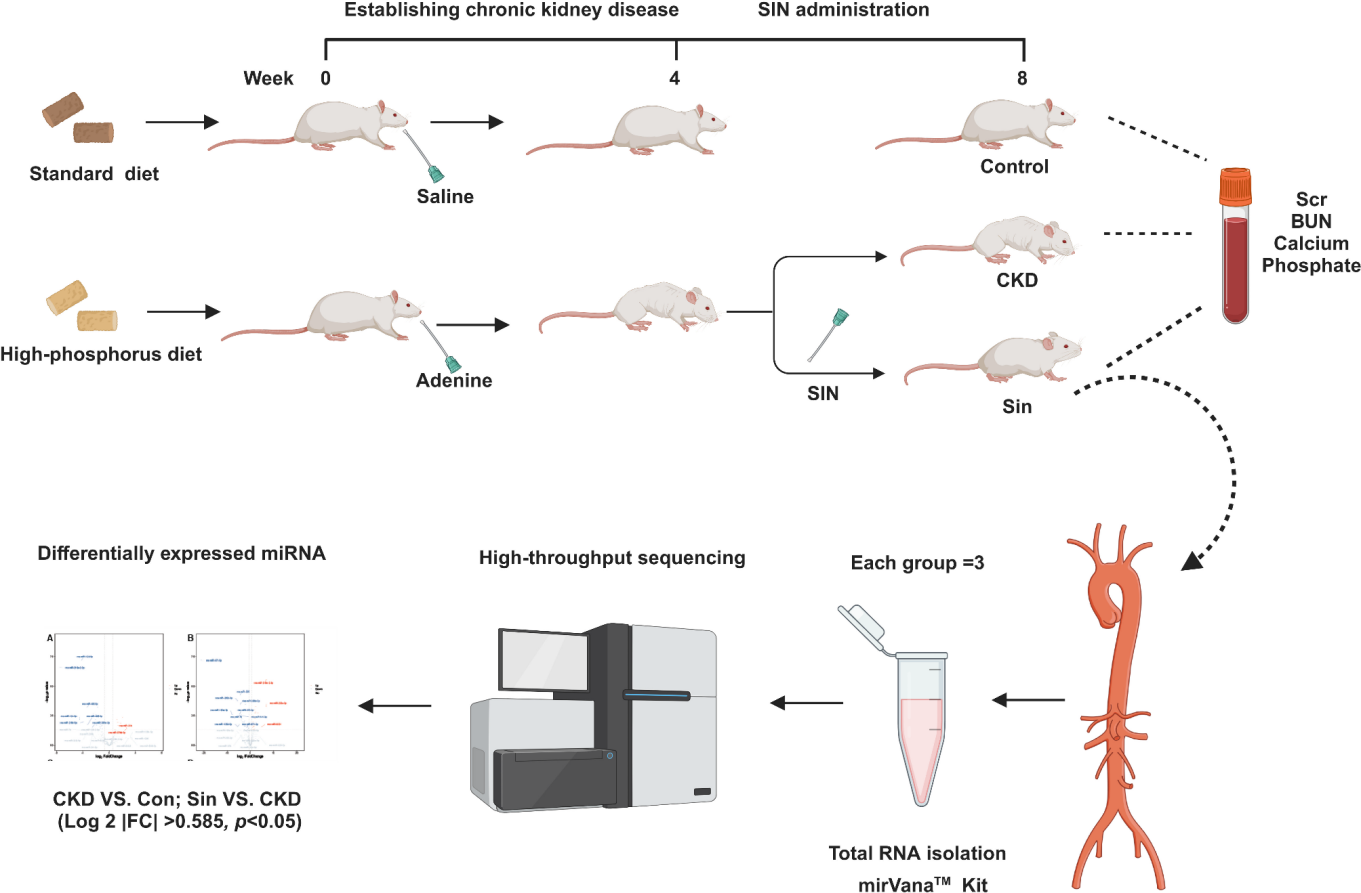
schematic diagram. Firstly, we established an adenine-induced chronic kidney disease uremic rat model. Subsequently, uremic rats were divided into CKD, SIN 20 mg and Sin. Finally, we collected blood and aortas. Three aortas in control group, CKD and Sin were used for miRNA-sequencing. *CKD* chronic kidney disease group, *SIN 20mg* sinomenine 20 mg/kg/d group, *Sin* sinomenine 40 mg/kg/d group.

**Fig. 2:**
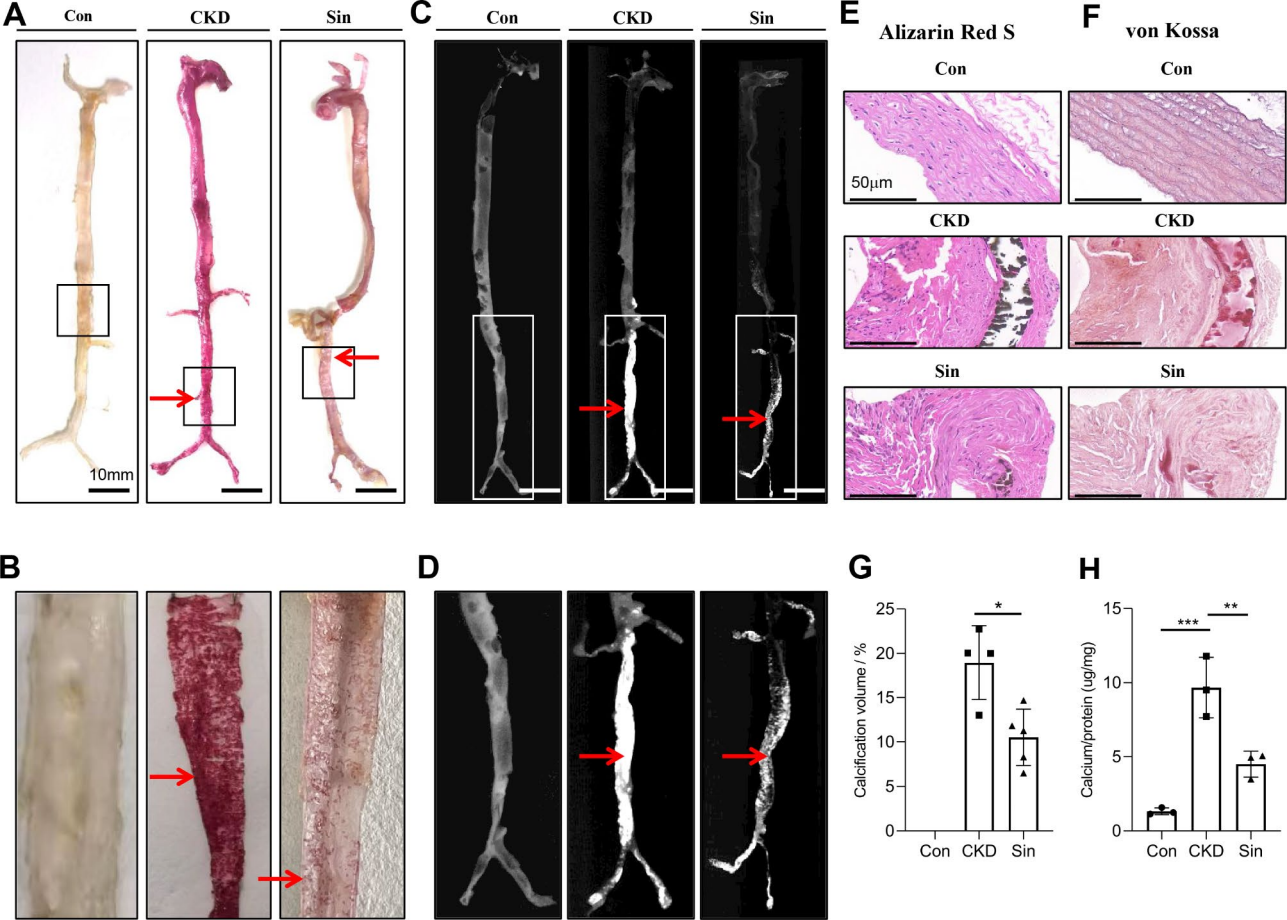
Sinomenine alleviated vascular calcification in adenine-induced uremic rats. (**A**) Representative images of translucent aorta from Con (control group), CKD (chronic kidney disease group) and Sin (sinomenine 40 mg/kg/d group). Representative samples were rendered translucent before alizarin red staining. Calcification depositions were stained positively in red. Calcification depositions in rat aortas significantly decreased in SIN compared with CKD. (**B**) Representative images of partial aorta from three corresponding group showed calcification depositions in rectangle area of (**A**). (**C**) Micro-CT images of aortas in Con, CKD and Sin. The micro-CT images are 3D-calcification-merged and calcification-extracted images. Calcification depositions in abdominal aortas significantly decreased in SIN compared with CKD. (**D**) The micro-CT images of partial aorta from three corresponding group showed calcification depositions in rectangle area of (**C**). Representative images showed von Kossa staining (**E**) and alizarin red staining (**F**) of cross-section of the abdominal aorta in three groups. (**G**) Quantification of calcification volume of aortas in CKD (*n* = 5) and Sin (*n* = 4) according to micro-CT. (**H**) Quantification of calcification depositions of aortas in Con, CKD and Sin normalized to µg Ca/mg protein weight. *n* = 3 per group. The arrowhead shows the calcification depositions. Scale bar: A and C = 10 mm; E and F = 50 μm. Error bars indicate standard deviations. For two groups comparison, Student’s t-test was used, while for comparisons > 2 groups, one-way analysis of variance was used. *P < 0.05, **P < 0.01, ***P < 0.001.

Firstly, we established a successful adenine-induced uremic rat model (Fig. 1). To evaluate macroscopic vascular calcification, optical operation rendered aortas transparent for alizarin red staining. Vascular calcification depositions in aortas from rats receiving 220 mg/kg/d adenine and high phosphate diet (CKD) were easily distinguished compared with aortas from control group (Con). Compared with CKD, administration of 40 mg/kg/d sinomenine (Sin) effectively alleviated calcified lesions in macroscopic view (Fig. 2A,B). The calcification crystallization of aorta in uremic rats was clearly observed in micro-CT images. Representative micro-CT images of calcification depositions in whole or partial aortas were consistent with macroscopic images of alizarin red staining (Fig. 2C,D). Sinomenine administration significantly reduced calcification according to quantification analysis of micro-CT (Fig. 2G). Similarly, we observed the same trend in microscopic images of aortas in von Kossa staining and alizarin red staining. The black or brown black calcification crystallization in von Kossa staining and red calcification crystallization in alizarin red staining were observed more in CKD and less in Sin (Fig. 2E,F). Calcium deposition quantification showed sinomenine inhibited calcification deposition in aortas (Fig. 2H). However, 20 mg/kg/d sinomenine administration didn’t inhibit vascular calcification significantly compared with CKD (Supplementary Figure S2). These results suggested that administration of 40 mg/kg/d sinomenine effectively alleviated vascular calcification in uremic rats.

### MiRNA-seq uncovered differentially expressed miRNAs in uremic rat aortas

**Fig. 3:**
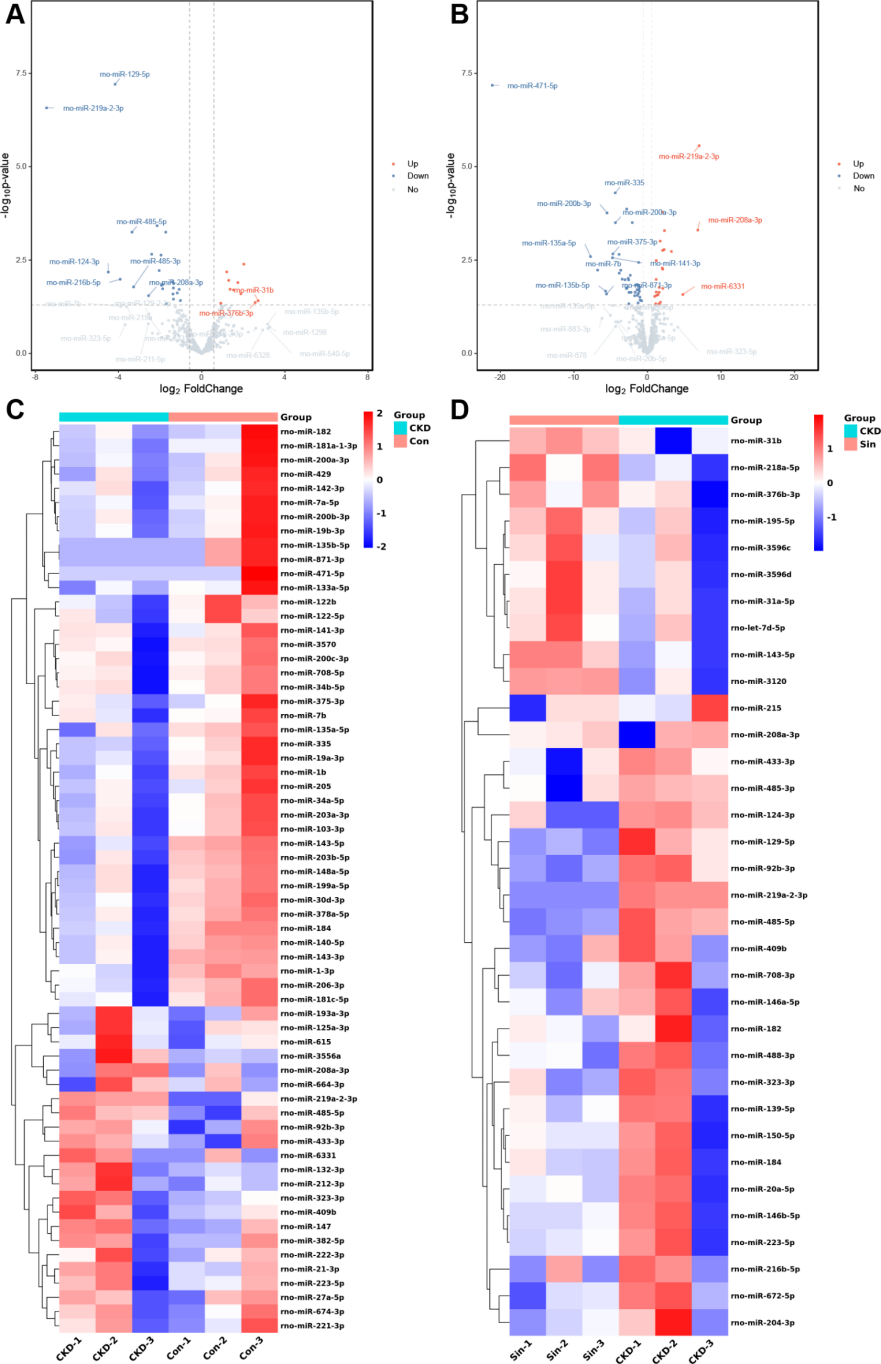
MiRNA-seq uncovered differentially expressed miRNAs in uremic rat aortas. (**A**) The volcano plots of differentially expressed miRNAs between Con and CKD in aortas. (**B**) The volcano plots of differentially expressed miRNAs between Sin and CKD in calcific aortas. (**C**) The heatmap of 64 differentially expressed miRNAs between Con and CKD. (**D**) The heatmap of 34 differentially expressed miRNAs between Sin and CKD. *n* = 3 per group. Log 2 |FC| > 0.585, *p* < 0.05. Blue represents lower relative expression, and red represents higher relative expression. *Con* control group, *CKD* chronic kidney disease group, *Sin* sinomenine 40 mg/kg/d group, *FC* fold change.

To reveal how sinomenine inhibited vascular calcification, we carry out miRNA-seq for further study. The volcano plots and heatmaps for miRNAs expressions are shown. Differentially expressed miRNAs were regulated by uremic vascular calcification and administration of sinomenine. In CKD compared with Con, 23 up-regulated miRNAs and 41 down-regulated miRNAs were screened. (Fig. 3A,D). In Sin compared with CKD, 10 up-regulated miRNAs and 24 down-regulated miRNAs were screened. Blue or red indicated down-regulated or up-regulated expression, respectively (Log 2 |FC| > 0.585, *p* < 0.05). These miRNAs were listed in (Supplementary Table S2–S5). We also provided volcano plots and hierarchical clustering of differentially expressed miRNAs with different fold change (Log 2 |FC| > 1.0, *q* < 0.05) (Supplementary Figure S3). These miRNAs were listed in Supplementary Table S6,S7.

### Venn diagrams screened overlaps of differentially expressed miRNAs

**Fig. 4:**
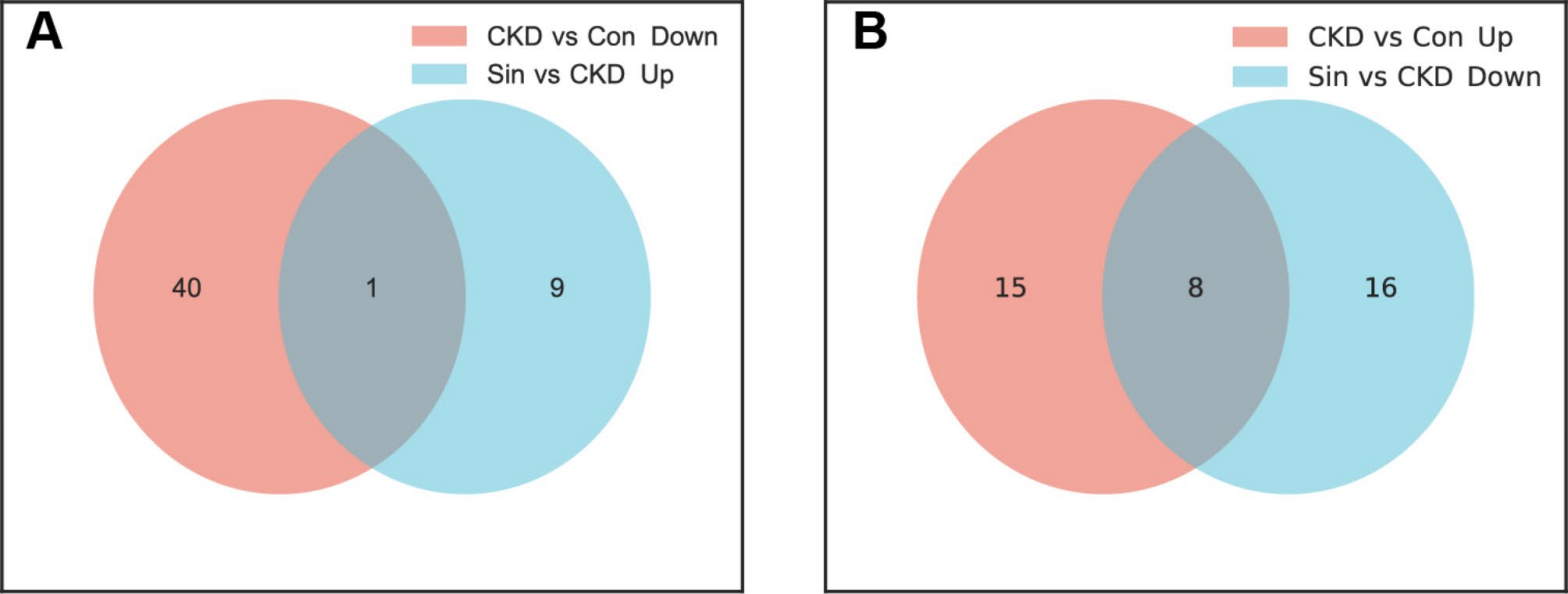
Venn diagrams screened overlaps of differentially expressed miRNAs. (**A**) Venn diagram showed 1 overlap miRNA (rno-miR-143-5p) which was down-regulated in CKD compared with Con, while up-regulated in Sin compared with CKD. (**B**) Venn diagram showed 8 overlaps miRNAs which were up-regulated in CKD compared with Con, while down-regulated in Sin compared with CKD. *Con* control group, *CKD* chronic kidney disease group, *Sin* sinomenine 40 mg/kg/d group.

In total, we selected 9 differentially expressed miRNAs for further gene prediction (Log 2 |FC| > 0.585, *p* < 0.05). Down-regulated in CKD compared with Con while up-regulated in Sin compared with CKD, 1 miRNA (rno-miR-143-5p) was predicted to alleviated vascular calcification (Fig. 4A). Up-regulated in CKD compared with Con while down-regulated in Sin compared with CKD, 8 miRNAs (including rno-miR-208a-3p, rno-miR-219a-2-3p, rno-miR-223-5p, rno-miR-323-3p, rno-miR-409b, rno-miR-433-3p, rno-miR-485-5p, rno-miR-92b-3p) were predicted to promote vascular calcification (Fig. 4B). These miRNAs were listed in (Tables 1 and 2).

**Table 1:** 9 screened miRNAs expressed in CKD vs. con.

Mature ID	Expression change	Sequence
rno-miR-143-5p	Down	GGUGCAGUGCUGCAUCUCUGG
rno-miR-208a-3p	Up	AUAAGACGAGCAAAAAGC
rno-miR-219a-2-3p	Up	AGAAUUGUGGCUGGACAUCUGU
rno-miR-223-5p	Up	CGUGUAUUUGACAAGCUGAGUUG
rno-miR-323-3p	Up	CACAUUACACGGUCGACCUCU
rno-miR-409b	Up	AGGGGUUCACCGAGCAACAUUCG
rno-miR-433-3p	Up	AUCAUGAUGGGCUCCUCGGUGU
rno-miR-485-5p	Up	AGAGGCUGGCCGUGAUGAAUUC
rno-miR-92b-3p	Up	UAUUGCACUCGUCCCGGCCUCC

**Table 2:** 9 screened miRNAs expressed in sin vs. CKD.

Mature ID	Expression change	Sequence
rno-miR-143-5p	Up	GGUGCAGUGCUGCAUCUCUGG
rno-miR-208a-3p	Down	AUAAGACGAGCAAAAAGC
rno-miR-219a-2-3p	Down	AGAAUUGUGGCUGGACAUCUGU
rno-miR-223-5p	Down	CGUGUAUUUGACAAGCUGAGUUG
rno-miR-323-3p	Down	CACAUUACACGGUCGACCUCU
rno-miR-409b	Down	AGGGGUUCACCGAGCAACAUUCG
rno-miR-433-3p	Down	AUCAUGAUGGGCUCCUCGGUGU
rno-miR-485-5p	Down	AGAGGCUGGCCGUGAUGAAUUC
rno-miR-92b-3p	Down	UAUUGCACUCGUCCCGGCCUCC

### Bioinformatic analysis of differentially expressed miRNAs

**Fig. 5:**
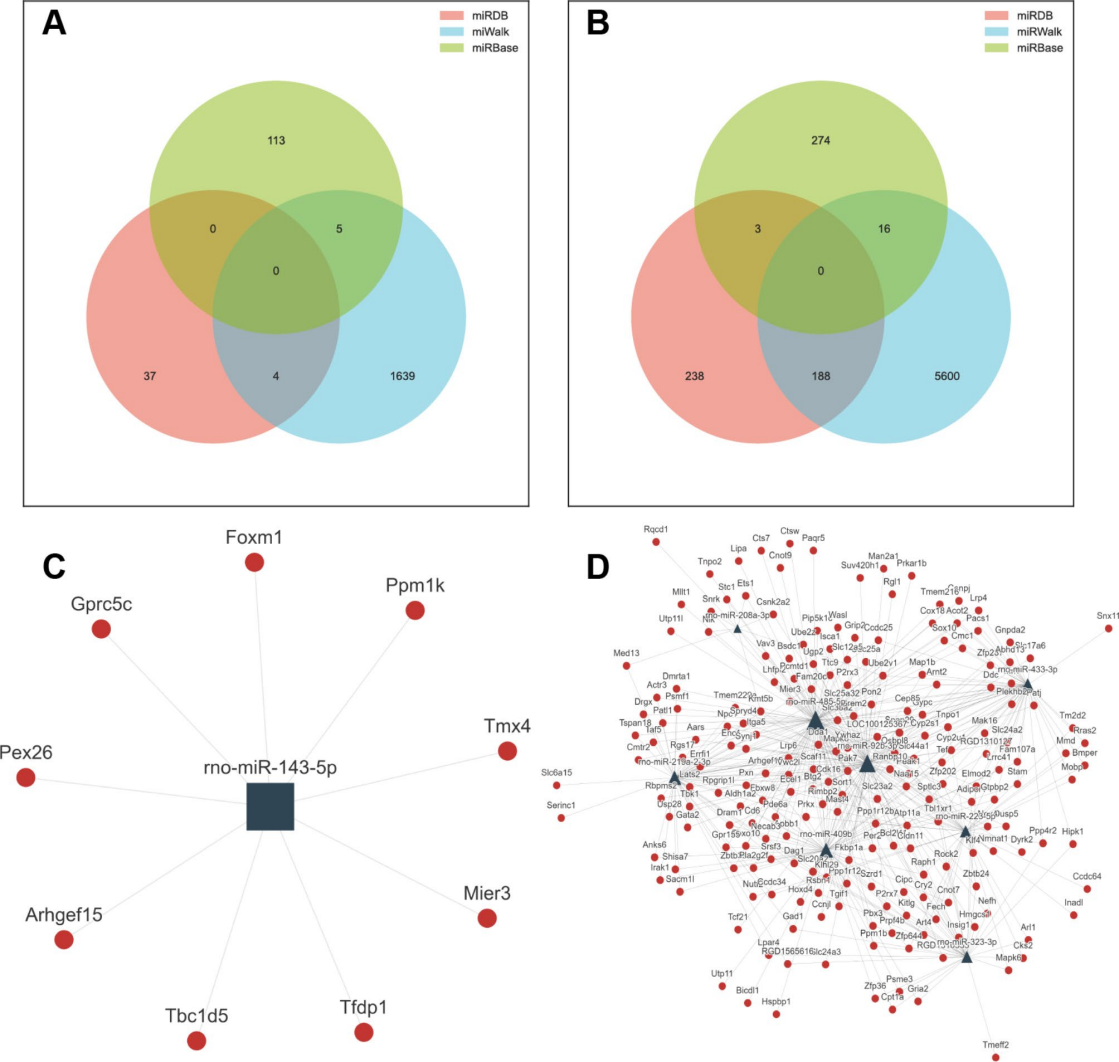
Bioinformatic analysis of differentially expressed miRNAs. (**A**) Venn diagram showed target gene prediction of rno-miR-143-5p in miRDB, miRWalk and miRBase. (**B**) Venn diagram showed target gene prediction of 8 screened miRNAs in miRDB, miRWalk and miRBase. (**C**) A proposed network of putative interactions between rno-miR-143-5p and mRNAs. (**D**) A proposed network of putative interactions between 8 screened miRNAs and mRNAs.

We further analyzed the target gene prediction of the 9 screened differentially expressed miRNAs in miRDB, miRWalk and miRBase databases (Fig. 5A,B). For rno-miR-143-5p, 8 overlap predicted target genes were listed in (Supplementary Table S8). For other screened 8 miRNAs, their overlap target genes were listed in (Supplementary Table S9, S10). To investigate the pathways of sinomenine against vascular calcification, we used the cytoscape networks to predict the relationships between differentially expressed miRNAs in CKD group and Sin group and their target genes. According to miRDB, miRWalk and miRBase databases, we used 9 differentially expressed miRNAs and their corresponding predicted target mRNAs to construct a miRNA-mRNA visualization network (Fig. 5C,D). Clustering GO (gene ontology) analysis and KEGG (Kyoto encyclopedia of genes and genomes) signaling pathways enrichment analysis were performed. According to the bioinformatic analysis, screened miRNAs were most related to regulation of cell proliferation and cell differentiation (Supplementary Figure S4–S7).

### Sinomenine and rno-mir-143-5p alleviated vascular calcification in A7R5 cells

**Fig. 6:**
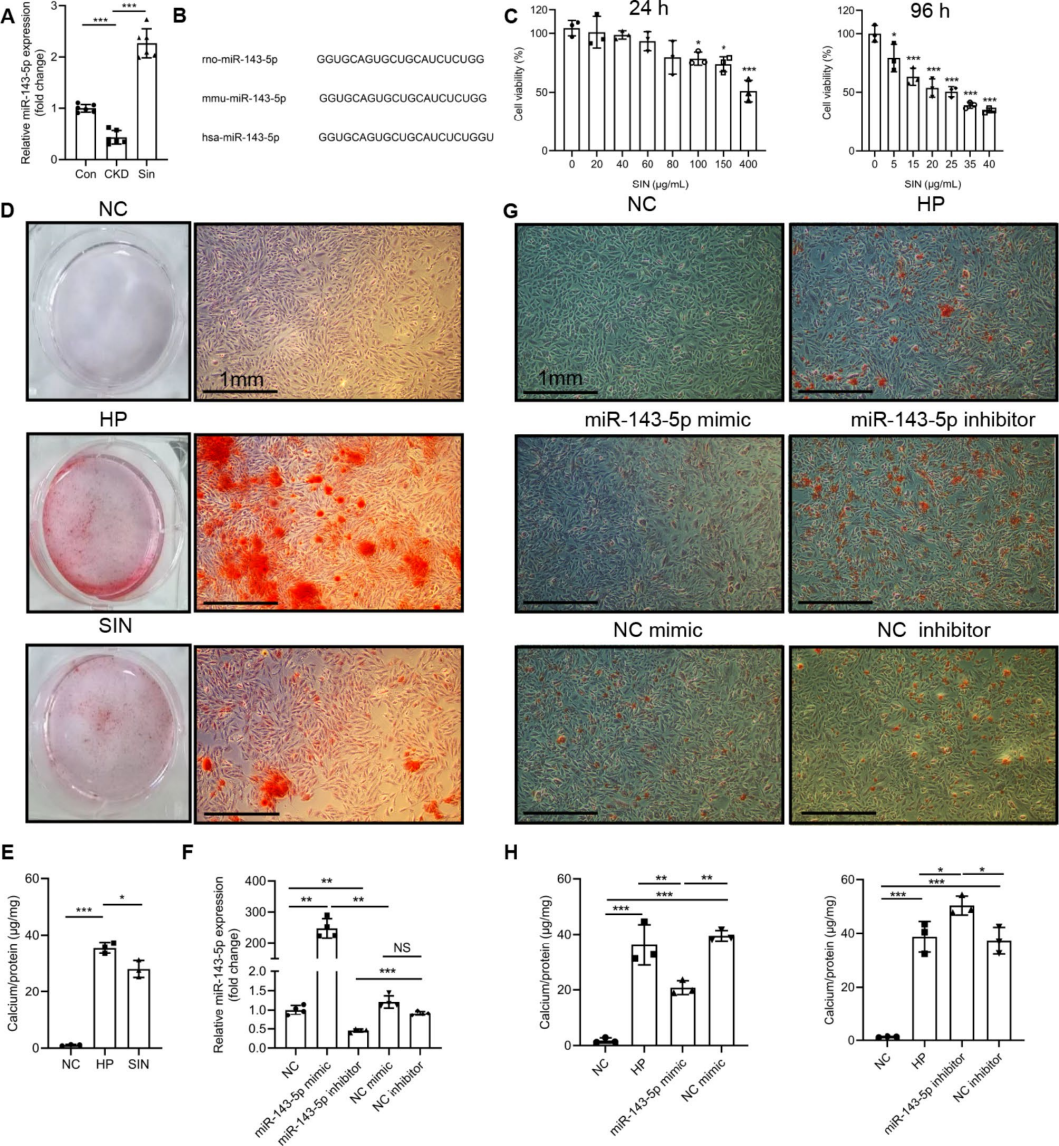
Sinomenine and rno-miR-143-5p alleviated vascular calcification in A7R5 cells. (**A**) Relative expression of rno-miR-143-5p in aortas tissue in Con, CKD and Sin, *n* = 6 per group. (**B**) Sequences of miR-143-5p in rat, mouse and human. (**C**) Sinomenine significantly inhibited A7R5 cells growth with a lower concentration at 96 h or a higher concentration at 24 h detected by CCK-8, compared with NC. *n* = 3 per group. (**D**) Alizarin red staining images showed HP significantly increased the calcium deposition in A7R5 cells compared with NC on seventh day, while SIN decreased A7R5 cells calcification. (**E**) Calcification depositions of (**D**) were quantified and normalized to µg Ca/mg protein weight. *n* = 3 per group. (**F**) Rno-miR-143-5p mimic or rno-miR-143-5p inhibitor was transfected to A7R5 cells. The expression of rno-miR-143-5p was increased or decreased compared with NC mimic or NC inhibitor, respectively. *n* = 4 per group. (**G**) Alizarin red staining showed rno-miR-143-5p mimic decreased calcification crystallization compared with NC mimic in high phosphate condition on seventh day, while rno-miR-143-5p inhibitor increased calcification crystallization compared with NC inhibitor in high phosphate condition. (**H**) Calcification depositions were quantified and normalized to µg Ca/mg protein weight. *n* = 3 per group. Scale bar: 1 mm. Error bars indicate standard deviations. One-way analysis of variance was used. Compared with control, *P < 0.05, **P < 0.01, ***P < 0.001. *CCK-8* cell counting kit-8, *NC* negative control, *HP* high phosphate, *SIN* 40 µg/mL sinomenine.

According to miRNA-seq, rno-miR-143-5p expression was lower in CKD compared with Con, while its expression was higher in Sin group compared with CKD. To confirm rno-miR-143-5p expression, we performed qRT-PCR in aortas tissue and the result was consistent with miRNA-seq (Fig. 6A). We also detected the other screened 8 miRNAs by qRT-PCR (Supplementary Figure S8). However, we found that the expressions of those 8 miRNAs in different groups were not statistically significant in our samples. Thus, we selected rno-miR-143-5p for further research. According to miRBase, the sequences of miR-143-5p in rat and mouse are the same while an additional uracil in human (Fig. 6B). While sinomenine administration alleviated vascular calcification in vivo, the effect of sinomenine administration on VSMC calcification in vitro remained unknown.

A7R5 cells were selected for further research. In order to explore the appropriate sinomenine concentration, we established a serial of concentrations of sinomenine administration on A7R5 cells and used cell counting kit-8 to analyze the cell viability. Sinomenine significantly inhibited A7R5 cells proliferation at 96 h (Fig. 6C). Subsequently, we established a serial of concentrations of sinomenine administration on A7R5 cells in high phosphate condition. We observed A7R5 cells calcification was inhibited by sinomenine in a dose-dependent manner, accompanied with A7R5 cells proliferation inhibition (Supplementary Figure S9). Microscopic alizarin red images showed 60 µg/mL and 100 µg/mL sinomenine significantly decreased A7R5 cells calcification in high phosphate condition, compared with 20 µg/mL sinomenine (Supplementary Figure S9). Simultaneously, sinomenine significantly inhibited A7R5 cells proliferation in a higher dose. Combined cell counting kit-8 results and this phenomenon, we selected 40 µg/mL sinomenine for calcification quantification and identified sinomenine effectively alleviated A7R5 cells calcification in high phosphate condition. Representative alizarin red staining images showed sinomenine reduced calcification crystallization in high phosphate condition both macroscopically and microscopically (Fig. 6D). Calcification depositions of A7R5 cells were quantified and were corresponding to alizarin red staining (Fig. 6E).

Moreover, the effect of rno-miR-143-5p on VSMC calcification remained unknown. Therefore, miR-143-5p mimic or inhibitor transfection in A7R5 cells was conducted, validated by qRT-PCR (Fig. 6F). Transfection of rno-miR-143-5p mimic effectively decreased A7R5 cells calcification, while rno-miR-143-5p inhibitor transfection increased A7R5 cells calcification in high phosphate condition. Representative alizarin red staining images of microscopic calcification depositions showed rno-miR-143-5p mimic decreased calcification crystallization compared with NC mimic in high phosphate condition (Fig. 6G). In return, rno-miR-143-5p inhibitor increased calcification crystallization compared with NC inhibitor in high phosphate condition (Fig. 6G). Calcification depositions were quantified and were corresponding to alizarin red staining (Fig. 6F).

Taken together, these results suggested that sinomenine and rno-miR143-5p mimic decreased A7R5 cell calcification, while rno-miR-143-5p inhibitor increased A7R5 cell calcification.

### Sinomenine alleviated vascular calcification via increasing mmu-mir-143-5p in MOVAS-1 cells

**Fig. 7:**
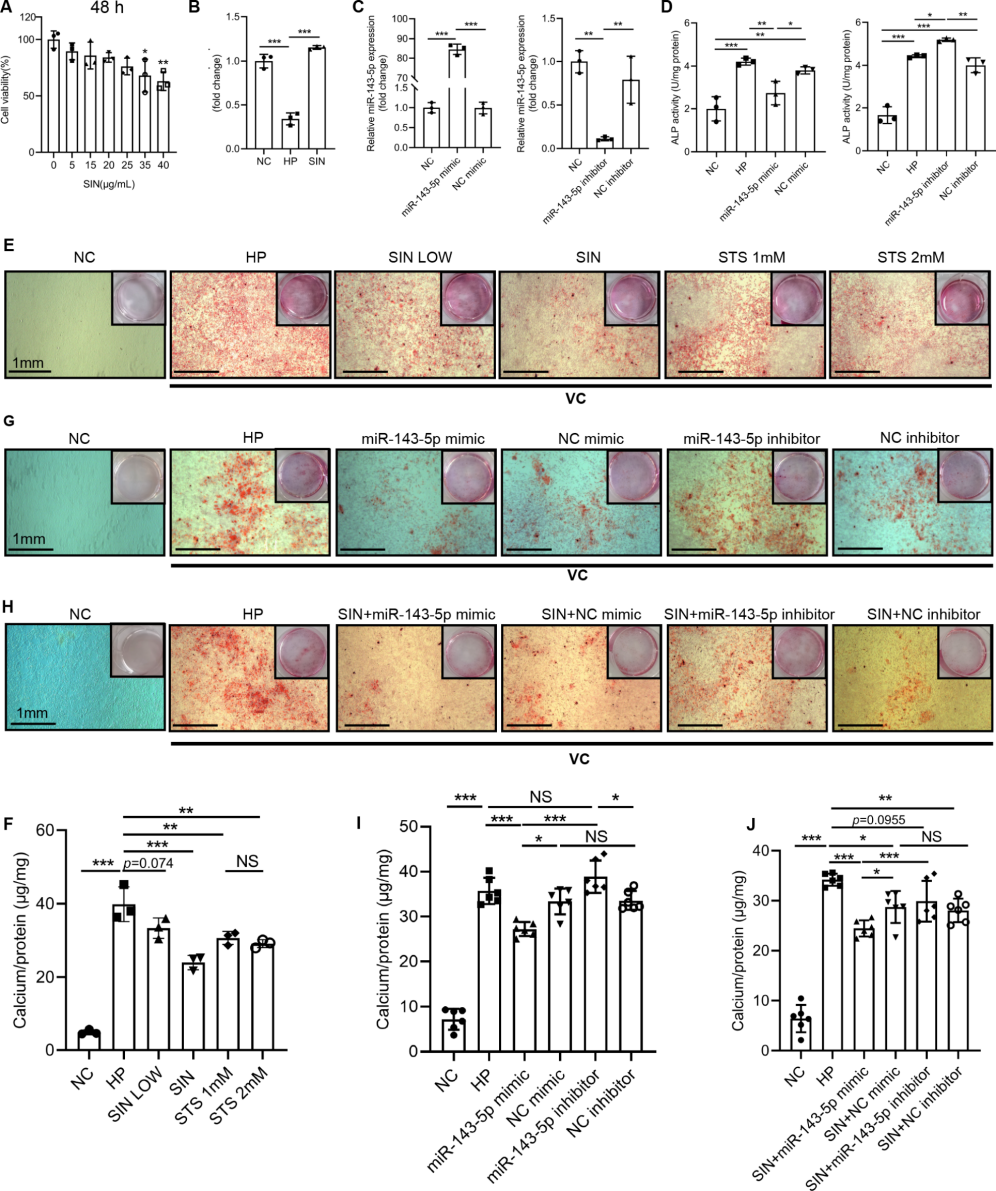
Sinomenine alleviated vascular calcification via increasing mmu-miR-143-5p in MOVAS-1 cells. (**A**) Sinomenine at 35 µg/mL significantly inhibited MOVAS-1 cells growth in 48 h detected by CCK-8, compared with NC. *n* = 3 per group. (**B**) Expression of mmu-miR-143-5p in MOVAS-1 cells decreased in HP compared with NC, while increased in SIN compared with HP on third day. *n* = 3 per group. (**C**) Mmu-miR-143-5p mimic or mmu-miR-143-5p inhibitor was transfected to MOVAS-1 cells. The expression of mmu-miR-143-5p was increased or decreased compared with NC mimic or NC inhibitor, respectively. *n* = 3 per group. (**D**) On seventh day, HP increased MOVAS-1 cells ALP activity compared with NC. Mmu-miR-143-5p mimic effectively decreased ALP activity compared with NC mimic in high phosphate condition, while mmu-miR-143-5p inhibitor increased ALP activity compared with NC inhibitor in high phosphate condition. *n* = 3 per group. (**E**) Alizarin Red S staining showed SIN at and STS significantly decreased calcification crystallization in high phosphate condition compared with HP on seventh day. (**F**) Calcification depositions were quantified and normalized to µg Ca/mg protein weight. There were not statistically significant between SIN LOW and HP. *n* = 3 per group. (**G**) Representative Alizarin Red S staining showed mmu-miR-143-5p mimic decreased calcification crystallization compared with NC mimic in high phosphate condition on seventh day, while mmu-miR-143-5p inhibitor increased calcification crystallization compared with NC inhibitor in high phosphate condition. (**H**) Representative Alizarin Red S staining showed sinomenine decreased calcification crystallization in NC mimic and NC inhibitor compared with HP. Moreover, mmu-miR-143-5p mimic decreased calcification crystallization compared with NC mimic and mmu-miR-143-5p inhibitor in high phosphate condition. (**I**,**J**) Calcification depositions were quantified and normalized to µg Ca/mg protein weight for (**G**) and (**H**), respectively. *n* = 6 per group. Scale bar: 1 mm. Error bars indicate standard deviations. One-way analysis of variance was used. *P < 0.05, **P < 0.01, ***P < 0.001. NS, not significant. *CCK-8* cell counting kit-8, *NC* negative control, *HP* high phosphate, *SIN* 40 µg/mL sinomenine, *SIN LOW* 20 µg/mL sinomenine, *STS* sodium thiosulfate, *VC* vascular calcification.

The effect of sinomenine administration on mouse VSMC calcification remained unknown. MOVAS-1 cells were selected for further research. Firstly, we established a serial of concentrations of sinomenine administration on MOVAS-1 cells and used cell counting kit-8 to analyze the cell viability. We identified 40 µg/mL sinomenine significantly inhibited MOVAS-1 cells proliferation at 48 h (Fig. 7A), while a higher concentration didn’t indicate a toxic effect on MOVAS-1 cells at 24 h (Supplementary Figure S10). We detected mmu-miR-143-5p expression in MOVAS-1 cells by qRT-PCR and confirmed mmu-miR-143-5p presented in MOVAS-1 cells. Moreover, mmu-miR-143-5p reduced in high phosphate condition while sinomenine reversed this process (Fig. 7B). Considering results of cell counting kit-8, we selected 20 µg/mL and 40 µg/mL sinomenine for further investigation. Evidences revealed that 40 µg/mL sinomenine effectively alleviated MOVAS-1 cells calcification in comparison to 20 µg/mL (Fig. 7E,F). Representative alizarin red staining images of calcification depositions showed 40 µg/mL sinomenine and sodium thiosulfate significantly reduced calcification crystallization in high phosphate condition both macroscopically and microscopically (Fig. 7E). Calcification depositions of MOVAS-1 cells were quantified and were corresponding to alizarin red staining (Fig. 7F). From above, evidences revealed 40 µg/mL sinomenine decreased MOVAS-1 cells calcification.

Moreover, the effect of mmu-miR-143-5p on mouse VSMC calcification remained unknown. Therefore, mmu-miR-143-5p mimic or mmu-miR-143-5p inhibitor was transfected into MOVAS-1 cells, validated by qRT-PCR (Fig. 7C). Subsequently, we detected ALP activity in MOVAS-1 cells and identified mmu-miR-143-5p mimic effectively decreased MOVAS-1 cell ALP activity, while mmu-miR-143-5p inhibitor increased MOVAS-1 cell ALP activity in high phosphate condition (Fig. 7D). Moreover, representative alizarin red staining images showed mmu-miR-143-5p mimic decreased calcification crystallization in high phosphate condition in comparison to NC mimic in low (Fig. 7G) and high (Supplementary Figure S11) magnification power of microscope. In return, mmu-miR-143-5p inhibitor increased calcification crystallization in high phosphate condition in comparison to NC inhibitor both microscopically and macroscopically (Fig. 7G and Supplementary Figure S11). Calcification depositions were quantified and were corresponding to alizarin red staining (Fig. 7I). Taken together, evidences revealed miR-143-5p mimic decreased MOVAS-1 cell calcification in comparison to NC mimic in high phosphate condition. On the contrary, miR-143-5p inhibitor increased MOVAS-1 cells calcification in comparison to NC inhibitor in high phosphate condition.

We further investigated regulation between sinomenine and miRNA transfection on MOVAS-1 cell calcification. Alizarin red staining images are represented in low (Fig. 7H) and high (Supplementary Figure S12) magnification power of microscope. Calcification depositions were quantified and were corresponding to alizarin red staining (Fig. 7J). Corresponding to Fig. 7E, 40 µg/mL sinomenine decreased MOVAS-1 cells calcification in NC mimic and NC inhibitor in high phosphate condition, compared with HP. Administrated with 40 µg/mL sinomenine in high phosphate condition, a stronger calcification reduction was found in mmu-miR-143-5p mimic in comparison to NC mimic (Fig. 7H,J, Supplementary Figure S12). From above, 40 µg/mL sinomenine decreased MOVAS-1 cells calcification regardless transfection of miR-143-5p mimic, NC mimic or NC inhibitor in high phosphate condition, compared with HP. Both administrated with 40 µg/mL sinomenine in high phosphate condition, miR-143-5p mimic decreased MOVAS-1 cells calcification in comparison to NC mimic. On the contrary, both administrated with 40 mg/mL sinomenine in high phosphate condition, the difference wasn’t significant between miR-143-5p inhibitor and NC inhibitor in high phosphate condition.

However, the difference between mmu-miR-143-5p inhibitor in high phosphate condition and HP was not significant. (Fig. 7G,I, Supplementary Figure S11). Interestingly, the difference was not significant but considerable between mmu-miR-143-5p inhibitor administrated with 40 µg/mL sinomenine in high phosphate condition and HP (*p* > 0.05) (Fig. 7H, J, Supplementary Figure S12). From our perspective, evidences indicated sinomenine decreased MOVAS-1 cells calcification in comparison to HP (Fig. 7E,F) while mmu-miR-143-5p inhibitor increased MOVAS-1 cells calcification in comparison to NC inhibitor in high phosphate condition (Fig. 7G,I). It is noteworthy that the difference was also not significant between mmu-miR-143-5p inhibitor and NC inhibitor, administrated with 40 µg/mL sinomenine in high phosphate condition. On the premise that sinomenine decreased MOVAS-1 cells calcification and mmu-miR-143-5p inhibitor increased MOVAS-1 cells calcification in comparison to NC inhibitor, we speculated sinomenine manifested a stronger calcification decrease than the calcification increases manifested by mmu-miR-143-5p inhibitor, otherwise the difference would be significant between mmu-miR-143-5p inhibitor and NC inhibitor both administrated with 40 µg/mL sinomenine in high phosphate condition. Mixed analyses of variance of Fig. 7I, J were consist with our inferences above (Supplementary Figure S13). Sinomenine decreased MOVAS-1 cells calcification regardless with or without miRNA mimic and miRNA inhibitor transfection in high phosphate condition, compared with those without administration of sinomenine (Supplementary Figure S13A,C). On the contrary, mmu-miR-143-5p inhibitor exerted a considerable antagonistic effect on vascular calcification protective effect of sinomenine, compared with NC inhibitor (Fig. 7H, J, Supplementary Figs. S12, S13). Briefly speaking, the protective effect of sinomenine on vascular calcification was stronger than the pro-calcification effect of mmu-miR-143-5p inhibitor.

Taken together, we inferred mmu-miR-143-5p reduction was not only a concomitant phenomenon in pro-calcification condition but also contribute to MOVAS-1 cells calcification. Correspondingly, mmu-miR-143-5p mimic and sinomenine decreased MOVAS-1 cells calcification in high phosphate condition. In conclusion, these evidences suggested sinomenine alleviated vascular calcification, which was attributed to miR-143-5p regulation partly.

### Exploring the plausibility of hsa-mir-143-5p as a circulating biomarker for uremic vascular calcification in clinical utility

**Fig. 8:**
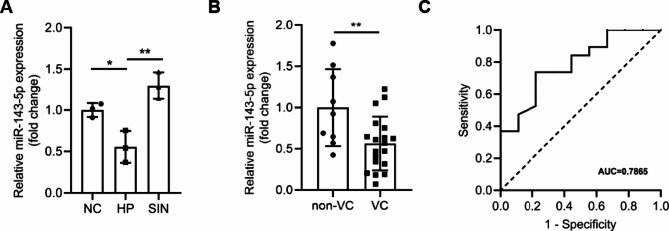
Exploring the plausibility of hsa-miR-143-5p as circulating biomarker for uremic vascular calcification in clinical utility. (**A**) Expression of has-miR-143-5p in T/G HA-VSMCs decreased in HP compared with NC, while increased in SIN compared with HP on third day. *n* = 3 per group. (**B**) Circulating hsa-miR-143-5p in plasma decreased in chronic kidney disease patients with vascular calcification (*n* = 19) compared with whom without vascular calcification (*n* = 9). (**C**) ROC curve based on relative expression of has-miR-143-5p among chronic kidney disease patients with or without vascular calcification, with AUROC value provided in the text. Error bars indicate standard deviations. For two groups comparison, Student’s t-test was used, while for comparisons > 2 groups, one-way analysis of variance was used. *P < 0.05, **P < 0.01. *NC* negative control, *HP* high phosphate, *SIN* 40 µg/mL sinomenine, *VC* vascular calcification, *ROC* receiver operating characteristic, *AUROC* area under receiver operating characteristic, *AUC* area under curve.

As mentioned above, we discovered decreased miR-143-5p expression in uremia vascular calcification concurrently within rat aorta and mouse VSMC. Furthermore, we observed reduction of has-miR-143-5p in T/G HA-VSMCs in high phosphate condition in vitro (Fig. 8A). Moreover, we prospectively enrolled 28 chronic kidney disease patients, among whom 9 (32.14%) did not have vascular calcification, while 19 (67.86%) had vascular calcification (Table 3). We subsequently examined the expression of circulating has-miR-143-5p by qRT-PCR among the chronic kidney disease cohort. The expression level of circulating hsa-miR-143-5p also decreased significantly in chronic kidney disease patients with vascular calcification, compared with those without vascular calcification (Fig. 8B). Furthermore, we built a ROC curve associated with vascular calcification in the validation cohort (Fig. 8C). Taken together, these findings highlighted the potential of hsa-miR-143-5p in predicting vascular calcification among uremia patients.

**Table 3:** Clinical features of chronic kidney disease patients (*n* = 28).

Features	No VC (*n* = 9)	VC (*n* = 19)	*P*-value
Demographic profile			
Ages (years)	56.3 ± 16.5	68.0 (56.0–72.0)	0.160
Gender (male %)	7 (78)	12 (63)	0.670
Duration of hemodialysis (years)	0 (0, 1.75)	0.4 (0, 2)	0.370
Comorbidity			
Diabetes mellitus (%)	0 (0)	6 (32)	0.136
Hypertension (%)	7 (78)	16 (84)	0.999
Heart failure (%)	3 (33)	7 (37)	0.999
Coronary artery disease (%)	1 (11)	4 (21)	0.999
Peripheral vascular disease (%)	1 (11)	2 (11)	0.999
Old cerebrovascular event (%)	0 (0)	5 (26)	0.144
Physical parameter			
Body mass index (kg/m^2^)	22.9 ± 3.3	23.2 ± 2.7	0.773
Systolic blood pressure (mmHg)	130.6 ± 29.5	144.7 ± 32.7	0.279
Diastolic blood pressure (mmHg)	81.9 ± 16.9	81.6 ± 18.5	0.972
Medication			
Aspirin (%)	1 (11)	5 (26)	0.630
Clopidogrel (%)	1 (11)	2 (11)	0.999
Statin (%)	0 (0)	5 (26)	0.144
Calcium-based phosphate binders (%)	7 (78)	13 (68.4)	0.999
Active vitamin D (%)	3 (33)	4 (21)	0.647
Laboratory parameters			
Hemoglobin (g/L)	88.0 ± 29.7	90.1 ± 22.0	0.839
Albumin (g/L)	38.0 ± 4.7	37.9 (36.6, 39.6)	0.838
Calcium (mmol/L)	1.8 ± 0.4	2.0 ± 0.2	0.147
Phosphate (mmol/L)	2.2 ± 0.9	2.0 ± 0.8	0.564
Ca-P product	48.9 ± 22.1	50.5 ± 18.8	0.856
iPTH (pg/mL)	375.2 ± 160.2	221.8 (163.5, 416.7)	0.172
Total cholesterol (mmol/L)	3.0 (2.4, 4.0)	3.3 (3.1, 3.9)	0.316
Triglyceride (mmol/L)	1.3 (0.8, 3.6)	1.6 ± 0.8	0.971
High density lipoprotein (mmol/L)	1.0 ± 0.3	1.0 ± 0.4	0.985
Low density lipoprotein (mmol/L)	1.5 ± 0.7	1.8 ± 0.8	0.403

Normally distributed continuous variables are presented as means ± standard deviations. For non-normally distributed variables, we presented data in median with 25% percentile and 75% percentile. Student’s t-test was used for normally distributed continuous variables to analyzed between-group differences. For comparing non-normally distributed variables of 2, we used Mann-Whitney U test. Fisher exact test was used for classified data.

*VC* vascular calcification, *iPTH* intact parathyroid hormone.

## Discussion

Sinomenine has been widely used in treatments of neuralgia and rheumatoid arthritis. The effect of sinomenine on inhibiting VSMCs phenotypic switching has been discovered recently, suggesting an inspiring application prospect in cardiovascular diseases^13^. Importantly, sinomenine decreased toll like receptor 4 (TLR4) and nuclear factor kappa B (NF-kB) expression, which played an crucial part in promoting VSMCs calcification in high phosphate conditions^14,15^. Sinomenine remarkably suppressed the development of atherosclerosis, which are closely related to intimal vascular calcification^16^. It inhibited metalloproteinase-2 (MMP-2) and metalloproteinase-9 (MMP-9), which were required for vascular calcification^17^. However, the direct effect of sinomenine on uremic vascular calcification has not been reported yet. In the present study, Sprague-Dawley rats with adenine and high-phosphorus diets were used to investigate the therapeutic effect of sinomenine. Our results indicated that sinomenine effectively inhibited vascular calcification in adenine-induced uremic rats. Moreover, sinomenine significantly inhibited VSMC calcification as sodium thiosulfate, which is a clinical drug for therapy against vascular calcification^18^. Meanwhile, we observed sinomenine significantly inhibited VSMCs proliferation in a dose-dependent manner attributing to VSMCs phenotypic switching regulation, indicating the potential application of sinomenine in cardiovascular diseases.

Recent studies have suggested that miRNAs appear to regulate the phenotypic switching of VSMCs in vascular calcification, such as miR-145a^19^. Therefore, we carried out miRNA-seq for aortas and revealed hundreds of differentially expressed miRNAs (Log 2 |FC| > 0.585, *p* < 0.05). We screened the overlap miRNAs for further investigation. We screened 8 miRNAs down-regulated by sinomenine (including rno-miR-208a-3p, rno-miR-219a-2-3p, rno-miR-223-5p, rno-miR-323-3p, rno-miR-409b, rno-miR-433-3p, rno-miR-485-5p, rno-miR-92b-3p), which were predicted to promote vascular calcification. We screened rno-miR-143-5p up-regulated by sinomenine, which was predicted to inhibit vascular calcification. We further screened predicted target genes of these miRNAs and used GO and KEGG pathway analyses^20^.

However, 8 miRNAs expression showed discrepancies between miRNA-seq and qRT-PCR results. We speculated that this might be due to individual difference of rat. Moreover, published researches also indicated potential and complicated roles of these miRNAs in vascular calcification. In VSMCs, miR-208a-3p inhibitor inhibited the phosphatidylinositol-3-kinase (PI3K)/AKT signaling pathway, which plays an important role in vascular calcification^21^. In human VSMCs, miR-223-5p mimics down-regulated insulin-like growth factor 1 receptor (IGF1R), and inhibition of IGF1R stimulated vascular calcification^22,23^. From patients with atherosclerosis or those with acute coronary syndromes, miR-323-3p was up-regulated in blood samples, indicating its function in promoting vascular calcification^24^. In diabetic patients with vascular calcification, miR-433-3p significantly decreased in comparison to the control group, especially^25^. In vitamin D3-induced vascular calcification rat model, miR-92b-3p was involved in vascular calcification^26^.

MiR-143-5p expression was consistent with our miRNA-seq prediction. MiR‑143‑3p and miR‑143‑5p derived from pre‑miRNA‑143. While there were numerous studies on the function of miR-143-3p in cardiovascular system, few researches concentrate on miR-143-5p. MiR-143-3p is required for VSMC acquisition of the contractile phenotype, by regulating the quiescent versus proliferative phenotype of smooth muscle cells^27^. Moreover, miR‑143‑3p regulated VSMC phenotype switching in combating atherosclerosis and vascular calcification^28,29^. We firstly demonstrated miR-143-5p also played a crucial role in vascular calcification. Pro-calcification condition down-regulated expression of miR-143-5p and downregulation of miR-143-5p, in turn, contributed to VSMCs calcification. In clinic, chronic kidney disease patients were found circulating miRNAs reduced in comparison to patients with normal renal function or mild renal impairment, such as miR-126-3p and miR-223-3p^30,31^. Furthermore, we demonstrated that chronic kidney disease patients with vascular calcification had a lower expression of circulating hsa-miR-143-5p than those who without vascular calcification. Thus, circulating hsa-miR-143-5p was supposed to predict vascular calcification in chronic kidney disease, which contribute to screen high risk vascular calcification population and provide certain diagnostic values for vascular calcification. However, our clinic cohort design limited the value of miR-143-5p application in predicting vascular calcification. Vascular calcification was a serious complication of chronic kidney disease. From our perspective, comparison between uremic patient with vascular calcification and uremic patient without vascular calcification would eliminate the interference of different renal function. However, we have ignored the effect of different vascular calcification severity and ignored circulating miR-143-5p expression in patients with normal renal function or mild renal impairment. Correspondingly, we demonstrated that miR-143-5p mimic decreased VSMC calcification in high phosphate condition, providing a valuable way to inhibit vascular calcification. In the future, it’s valuable to explore the effect of miR-143-5p agomir in vascular calcification animal model.

Individual miRNAs modulate collections of target mRNA expression, thereby governing complex biological processes^32^. MiRNA-143-5p promoted the phenotype conversion, proliferation and migration of VSMCs by targeting hypoxia inducible factor-1a^33^. Elevation of hypoxia inducible factor-1a level is associated with vascular calcification in VSMCs and in serum from non-dialysis chronic kidney disease patients with vascular calcification^34^. We also screened predicted target genes of miR-143-5p, such as ARHGEF15 and GPRC5C. Rho guanine nucleotide exchange factor 15 (ARHGEF15) mediated activation of cell division cycle 42, which promoted vascular calcification in CKD^35,36^. G-protein coupled receptor 5 C (GPRC5C) was elevated in dedifferentiating VSMCs and essential for osteoblast differentiation^37,38^. The miRNA-mRNA interaction mechanism should be illustrated in the future, which can provide more evidences in vitro and *in vivo.*

Taken together, we inferred miR-143-5p reduction was not only a concomitant phenomenon in pro-calcification condition but also contribute to VSMCs calcification. The protective effect of sinomenine on vascular calcification was stronger than the pro-calcification effect of mmu-miR-143-5p inhibitor. From our perspective, the regulation between miR-143-5p and sinomenine couldn’t fully illuminate the protective effect of sinomenine, indicating unrevealed mechanisms existed underlying sinomenine as we discussed above^13–17^. In conclusion, sinomenine effectively alleviated vascular calcification, which was attributed to miR-143-5p regulation partly.

## Conclusion

This was the first study to reveal sinomenine inhibited vascular calcification, we demonstrated that sinomenine alleviated vascular calcification in adenine-induced uremic rats. To reveal miRNAs involved in vascular calcification and modified by sinomenine, we detected the miRNAs profile of uremic rats’ calcific aortas administrated with or without sinomenine. Moreover, we screened 9 differentially expressed miRNAs modified by sinomenine and predicted their potential target genes. MiR-143-5p was validated and selected for further research. In addition, we demonstrated sinomenine effectively alleviated A7R5 cell and MOVAS-1 cell calcification. By miR-143-5p mimic and miR-143-5p transfection, we demonstrated sinomenine alleviated vascular calcification via increasing miR-143-5p in VSMCs. Finally, we demonstrated the expression level of circulating miR-143-5p declined among chronic kidney disease patients with vascular calcification. MiR-143-5p was supposed to a biomarker for uremia vascular calcification.

## Electronic supplementary material

Below is the link to the electronic supplementary material.

Supplementary Material 1

Supplementary Material 2

Supplementary Material 3

Supplementary Material 4

Supplementary Material 5

Supplementary Material 6

Supplementary Material 7

Supplementary Material 8

Supplementary Material 9

Supplementary Material 10

Supplementary Material 11

Supplementary Material 12

Supplementary Material 13

Supplementary Material 14

Supplementary Material 15

## Data Availability

All data generated or analysed during this study are included in this published article and its supplementary information files.

## Abbreviations

VSMCs: Vascular smooth muscle cells
mRNA: messenger RNA
miRNA: microRNA
miRNA-seq: miRNA-sequence
Con: Control group
CKD: Chronic kidney disease group
Sin: Sinomenine group
CCK-8: Cell counting kit-8
STS: Sodium thiosulfate
ALP: Alkaline phosphatase
qRT-PCR: Quantitative reverse transcription polymerase chain reaction
GO: Gene ontology
KEGG: Kyoto encyclopedia of genes and genomes
NC: Negative control
LP: Low phosphate
HP: High phosphate
SIN LOW: 20 µg/mL sinomenine
SIN: 40 µg/mL sinomenine
VC: Vascular calcification
TLR4: Toll like receptor 4
NF-κB: Nuclear factor kappa B
MMP: Metalloproteinase
PI3K: Phosphatidylinositol-3-kinase
IGF1R: Insulin-like growth factor 1 receptor
ARHGEF15: Rho guanine nucleotide exchange factor 15
GPRC5C: G-protein coupled receptor 5 C
