# Supplementary material for: Sinomenine attenuates uremia vascular calcification by miR-143-5p
Source: Sci Rep. 2025 Jan 13;15:1798. doi: 10.1038/s41598-025-86055-2 (PMC11730593; doi:10.1038/s41598-025-86055-2)
Supplement: Supplementary file 14 — Supplementary Material 14 [file 41598_2025_86055_MOESM14_ESM.docx]

**Supplementary Table 1** Primer for miRNA

| **miRNA** | **Sequence** |
| --- | --- |
| rno-miR-143-5p | 5’-GGTGCAGTGCTGCATCTCTGG-3’ |
| rno-miR-208a-3p | 5’-ATAAGACGAGCAAAAAGC-3’ |
| rno-miR-219a-2-3p | 5’-AGAATTGTGGCTGGACATCTGT-3’ |
| rno-miR-223-5p | 5’-CGTGTATTTGACAAGCTGAGTTG-3’ |
| rno-miR-323-3p | 5’-CACATTACACGGTCGACCTCT-3’ |
| rno-miR-409b | 5’-AGGGGTTCACCGAGCAACATTCG-3’ |
| rno-miR-433-3p | 5’-ATCATGATGGGCTCCTCGGTGT-3’ |
| rno-miR-485-5p | 5’-AGAGGCTGGCCGTGATGAATTC-3’ |
| rno-miR-92b-3p | 5’-TATTGCACTCGTCCCGGCCTCC-3’ |
| mmu-miR-143-5p | 5’-GGTGCAGTGCTGCATCTCTGG-3’ |
| hsa-miR-143-5p | 5’-GGTGCAGTGCTGCATCTCTGGT-3’ |
| has-miR-486-5p | 5’-TCCTGTACTGAGCTGCCCCGAG-3’ |

**Supplementary Table 2** 23 up-regulated miRNAs expressed in CKD vs. Con (Log 2 |FC| > 0.585, *p* < 0.05).

| **Mature ID** | **Expression change** | **Sequence** |
| --- | --- | --- |
| rno-miR-219a-2-3p | Up | AGAATTGTGGCTGGACATCTGT |
| rno-miR-132-3p | Up | TAACAGTCTACAGCCATGGTCG |
| rno-miR-208a-3p | Up | ATAAGACGAGCAAAAAGC |
| rno-miR-212-3p | Up | TAACAGTCTCCAGTCACGGCCA |
| rno-miR-147 | Up | GTGTGCGGAAATGCTTCTGCTA |
| rno-miR-323-3p | Up | CACATTACACGGTCGACCTCT |
| rno-miR-92b-3p | Up | TATTGCACTCGTCCCGGCCTCC |
| rno-miR-485-5p | Up | AGAGGCTGGCCGTGATGAATTC |
| rno-miR-222-3p | Up | AGCTACATCTGGCTACTGGGT |
| rno-miR-409b | Up | AGGGGTTCACCGAGCAACATTCG |
| rno-miR-3556a | Up | CGATTTCAGATGGTGCTA |
| rno-miR-674-3p | Up | CACAGCTCCCATCTCAGAACAA |
| rno-miR-125a-3p | Up | ACAGGTGAGGTTCTTGGGAGCC |
| rno-miR-433-3p | Up | ATCATGATGGGCTCCTCGGTGT |
| rno-miR-27a-5p | Up | AGGGCTTAGCTGCTTGTGAGCA |
| rno-miR-193a-3p | Up | AACTGGCCTACAAAGTCCCAGT |
| rno-miR-6331 | Up | CTTTGGTGGCTTAGTTCTTTGTGC |
| rno-miR-382-5p | Up | GAAGTTGTTCGTGGTGGATTCG |
| rno-miR-21-3p | Up | CAACAGCAGTCGATGGGCTGTC |
| rno-miR-221-3p | Up | AGCTACATTGTCTGCTGGGTTTC |
| rno-miR-615 | Up | GGGGGTCCCCGGTGCTCGGATC |
| rno-miR-223-5p | Up | CGTGTATTTGACAAGCTGAGTTG |
| rno-miR-664-3p | Up | TATTCATTTACTCCCCAGCCTA |

**Supplementary Table 3** 41 down-regulated miRNAs expressed in CKD vs. Con (Log 2 |FC| > 0.585, *p* < 0.05).

| **Mature ID** | **Expression change** | **Sequence** |
| --- | --- | --- |
| rno-miR-471-5p | Down | TACGTAGTATAGTGCTTTTCAC |
| rno-miR-335 | Down | TCAAGAGCAATAACGAAAAATGT |
| rno-miR-1b | Down | TGGAATGTAAAGAAGTATGTAT |
| rno-miR-200b-3p | Down | TAATACTGCCTGGTAATGATGAC |
| rno-miR-184 | Down | TGGACGGAGAACTGATAAGGGT |
| rno-miR-200a-3p | Down | TAACACTGTCTGGTAACGATGT |
| rno-miR-375-3p | Down | TTTGTTCGTTCGGCTCGCGTGA |
| rno-miR-429 | Down | TAATACTGTCTGGTAATGCCGT |
| rno-miR-135a-5p | Down | TATGGCTTTTTATTCCTATGTGA |
| rno-miR-141-3p | Down | TAACACTGTCTGGTAAAGATGG |
| rno-miR-143-5p | Down | GGTGCAGTGCTGCATCTCTGG |
| rno-miR-7b | Down | TGGAAGACTTGTGATTTTGTTGT |
| rno-miR-3570 | Down | GGTACAATCAACGGTCGATGGT |
| rno-miR-200c-3p | Down | TAATACTGCCGGGTAATGATG |
| rno-miR-19a-3p | Down | TGTGCAAATCTATGCAAAACTGA |
| rno-miR-122b | Down | AACACCATTGTCACACTCCA |
| rno-miR-133a-5p | Down | AGCTGGTAAAATGGAACCAAAT |
| rno-miR-182 | Down | TTTGGCAATGGTAGAACTCACACCG |
| rno-miR-181a-1-3p | Down | ACCATCGACCGTTGATTGTACC |
| rno-miR-203a-3p | Down | GTGAAATGTTTAGGACCACTAG |
| rno-miR-34a-5p | Down | TGGCAGTGTCTTAGCTGGTTGT |
| rno-miR-30d-3p | Down | CTTTCAGTCAGATGTTTGCTGC |
| rno-miR-140-5p | Down | CAGTGGTTTTACCCTATGGTAG |
| rno-miR-142-3p | Down | TGTAGTGTTTCCTACTTTATGGA |
| rno-miR-708-5p | Down | AAGGAGCTTACAATCTAGCTGGG |
| rno-miR-148a-5p | Down | AAAGTTCTGAGACACTCTGACTC |
| rno-miR-103-3p | Down | AGCAGCATTGTACAGGGCTATGA |
| rno-miR-135b-5p | Down | TATGGCTTTTCATTCCTATGTGA |
| rno-miR-378a-5p | Down | CTCCTGACTCCAGGTCCTGTGT |
| rno-miR-205 | Down | TCCTTCATTCCACCGGAGTCTGT |
| rno-miR-19b-3p | Down | TGTGCAAATCCATGCAAAACTGA |
| rno-miR-122-5p | Down | TGGAGTGTGACAATGGTGTTTG |
| rno-miR-206-3p | Down | TGGAATGTAAGGAAGTGTGTGG |
| rno-miR-871-3p | Down | TGACTGGCACCATACTGGATAA |
| rno-miR-181c-5p | Down | AACATTCAACCTGTCGGTGAGT |
| rno-miR-7a-5p | Down | TGGAAGACTAGTGATTTTGTTGT |
| rno-miR-203b-5p | Down | AGTGGTCCTAAACATTTCAC |
| rno-miR-143-3p | Down | TGAGATGAAGCACTGTAGCTCA |
| rno-miR-199a-5p | Down | CCCAGTGTTCAGACTACCTGTTC |
| rno-miR-1-3p | Down | TGGAATGTAAAGAAGTGTGTAT |
| rno-miR-34b-5p | Down | AGGCAGTGTAATTAGCTGATTGT |

**Supplementary Table 4** 10 up-regulated miRNAs expressed in Sin vs. CKD (Log 2 |FC| > 0.585, *p* < 0.05).

| **Mature ID** | **Expression change** | **Sequence** |
| --- | --- | --- |
| rno-miR-31a-5p | Up | AGGCAAGATGCTGGCATAGCTG |
| rno-miR-143-5p | Up | GGTGCAGTGCTGCATCTCTGG |
| rno-miR-218a-5p | Up | TTGTGCTTGATCTAACCATGT |
| rno-let-7d-5p | Up | AGAGGTAGTAGGTTGCATAGTT |
| rno-miR-195-5p | Up | TAGCAGCACAGAAATATTGGC |
| rno-miR-3596d | Up | CTATACAATCTACTACCTCA |
| rno-miR-3596c | Up | ACTATACAACCTCCTACCTCA |
| rno-miR-31b | Up | CTATGCCAGCATCTTGCCT |
| rno-miR-376b-3p | Up | ATCATAGAGGAACATCCACTT |
| rno-miR-3120 | Up | CACAGCAAGTGTAGACAGGCA |

**Supplementary Table 5** 24 down-regulated miRNAs expressed in Sin vs. CKD (Log 2 |FC| > 0.585, *p* < 0.05).

| **Mature ID** | **Expression change** | **Sequence** |
| --- | --- | --- |
| rno-miR-129-5p | Down | CTTTTTGCGGTCTGGGCTTGC |
| rno-miR-219a-2-3p | Down | AGAATTGTGGCTGGACATCTGT |
| rno-miR-92b-3p | Down | TATTGCACTCGTCCCGGCCTCC |
| rno-miR-485-5p | Down | AGAGGCTGGCCGTGATGAATTC |
| rno-miR-708-3p | Down | CAACTAGACTGTGAGCTTCTAG |
| rno-miR-433-3p | Down | ATCATGATGGGCTCCTCGGTGT |
| rno-miR-146b-5p | Down | TGAGAACTGAATTCCATAGGCTGT |
| rno-miR-323-3p | Down | CACATTACACGGTCGACCTCT |
| rno-miR-124-3p | Down | TAAGGCACGCGGTGAATGCC |
| rno-miR-216b-5p | Down | AAATCTCTGCAGGCAAATGTGA |
| rno-miR-223-5p | Down | CGTGTATTTGACAAGCTGAGTTG |
| rno-miR-20a-5p | Down | TAAAGTGCTTATAGTGCAGGTAG |
| rno-miR-672-5p | Down | TGAGGTTGGTGTACTGTGTGTGA |
| rno-miR-215 | Down | ATGACCTATGATTTGACAGACA |
| rno-miR-485-3p | Down | CATACACGGCTCTCCTCTCTTC |
| rno-miR-139-5p | Down | TCTACAGTGCACGTGTCTCCAG |
| rno-miR-409b | Down | AGGGGTTCACCGAGCAACATTCG |
| rno-miR-150-5p | Down | TCTCCCAACCCTTGTACCAGTG |
| rno-miR-182 | Down | TTTGGCAATGGTAGAACTCACACCG |
| rno-miR-184 | Down | TGGACGGAGAACTGATAAGGGT |
| rno-miR-208a-3p | Down | ATAAGACGAGCAAAAAGC |
| rno-miR-204-3p | Down | GCTGGGAAGGCAAAGGGACGTT |
| rno-miR-146a-5p | Down | TGAGAACTGAATTCCATGGGTT |
| rno-miR-488-3p | Down | TTGAAAGGCTGTTTCTTGGTC |

**Supplementary Table 6** 11 differential miRNAs expressed in CKD vs. Control (Log 2 |FC| > 1.0, *q* < 0.05).

| **Mature ID** | **Expression change** | **Sequence** |
| --- | --- | --- |
| rno-miR-132-3p | Up | TAACAGTCTACAGCCATGGTCG |
| rno-miR-147 | Up | GTGTGCGGAAATGCTTCTGCTA |
| rno-miR-208a-3p | Up | ATAAGACGAGCAAAAAGC |
| rno-miR-212-3p | Up | TAACAGTCTCCAGTCACGGCCA |
| rno-miR-219a-2-3p | Up | AGAATTGTGGCTGGACATCTGT |
| rno-miR-335 | Down | TCAAGAGCAATAACGAAAAATGT |
| rno-miR-471-5p | Down | TACGTAGTATAGTGCTTTTCAC |
| rno-miR-184 | Down | TGGACGGAGAACTGATAAGGGT |
| rno-miR-1b | Down | TGGAATGTAAAGAAGTATGTAT |
| rno-miR-200a-3p | Down | TAACACTGTCTGGTAACGATGT |
| rno-miR-200b-3p | Down | TAATACTGCCTGGTAATGATGAC |

**Supplementary Table 7** 5 down-regulated miRNAs in Sin compared to CKD (Log 2 |FC| > 1.0, *q* < 0.05).

| **Mature ID** | **Expression change** | **Sequence** |
| --- | --- | --- |
| rno-miR-129-5p | Down | CTTTTTGCGGTCTGGGCTTGC |
| rno-miR-219a-2-3p | Down | AGAATTGTGGCTGGACATCTGT |
| rno-miR-485-5p | Down | AGAGGCTGGCCGTGATGAATTC |
| rno-miR-708-3p | Down | CAACTAGACTGTGAGCTTCTAG |
| rno-miR-92b-3p | Down | TATTGCACTCGTCCCGGCCTCC |

**Supplementary Table 8** Overlapping target genes prediction of rno-miR-143-5p

| **Target Gene** | **Official Full Name** | **Genbank** | **overlapping** |
| --- | --- | --- | --- |
| Tbc1d5 | TBC1 domain family, member 5 | NM_001134762 | miRBase and miRWalk |
| Arhgef15 | Rho guanine nucleotide exchange factor 15 | NM_001105789 | miRBase and miRWalk |
| Pex26 | peroxisomal biogenesis factor 26 | NM_001106618.2 | miRBase and miRWalk |
| Gprc5c | G protein-coupled receptor class C group 5 member C | XM_017597153.1 | miRBase and miRWalk |
| Foxm1 | forkhead box M1 | XM_006237417.3 | miRBase and miRWalk |
| Ppm1k | protein phosphatase, Mg2+/Mn2+ dependent 1K | NM_001107863 | miRDB and miRWalk |
| Tmx4 | thioredoxin related transmembrane protein 4 | NM_001100529 | miRDB and miRWalk |
| Mier3 | MIER family member 3 | NM_001168000.1 | miRDB and miRWalk |
| Tfdp1 | transcription factor Dp-1 | NM_001025718 | miRDB and miRWalk |

**Supplementary Table 9** 19 overlapping target genes prediction of 8 miRNAs

| **Target Gene** | **Official Full Name** | **Genbank** | **overlapping** |
| --- | --- | --- | --- |
| Cyp2u1 | cytochrome P450 family 2 subfamily U member 1 | NM_001024779.1 | miRBase and miRWalk |
| Cyp2u1 | cytochrome P450 family 2 subfamily U member 1 | XM_006233310.3 | miRBase and miRWalk |
| Pxn | paxillin | NM_001012147.1 | miRBase and miRWalk |
| Pde6a | phosphodiesterase 6A | NM_001107386.1 | miRBase and miRWalk |
| Arnt2 | aryl hydrocarbon receptor nuclear translocator 2 | NM_012781.3 | miRBase and miRWalk |
| Mak16 | MAK16 homolog | NM_001014002.1 | miRBase and miRWalk |
| Cts7 | cathepsin 7 | NM_001106099.1 | miRBase and miRWalk |
| Fbxo10 | F-box protein 10 | XM_342829.8 | miRBase and miRWalk |
| Apbb1 | amyloid beta precursor protein binding family B member 1 | NM_080478.1 | miRBase and miRWalk |
| Fam107a | family with sequence similarity 107 member A | NM_001025129.1 | miRBase and miRWalk |
| Mast4 | microtubule associated serine/threonine kinase family member 4 | XM_017591364.1 | miRBase and miRWalk |
| Zbtb24 | zinc finger and BTB domain containing 24 | NM_001098667.1 | miRBase and miRWalk |
| Hoxd4 | homeo box D4 | NM_001105885.1 | miRBase and miRWalk |
| Mast4 | microtubule associated serine/threonine kinase family member 4 | XM_008760704.2 | miRBase and miRWalk |
| P2rx3 | purinergic receptor P2X 3 | NM_001270621.1 | miRBase and miRWalk |
| Slc20a2 | solute carrier family 20 member 2 | NM_017223.2 | miRBase and miRWalk |
| Ttc9 | tetratricopeptide repeat domain 9 | NM_001134731.1 | miRBase and miRDB |
| Stc1 | stanniocalcin 1 | NM_031123.2 | miRBase and miRDB |
| Sptlc3 | serine palmitoyltransferase, long chain base subunit 3 | NM_001106517.1 | miRBase and miRDB |

**Supplementary Table 10** 188 target genes prediction of 8 miRNAs between miRBD and miRWalk

| **Target Gene** | **Genbank** |
| --- | --- |
| Rgs17 | NM_001107459.1 |
| Gnpda2 | NM_001106005.1 |
| Zfp287 | NM_001107008.2 |
| Man2a1 | NM_012979.2 |
| Map1b | NM_019217.1 |
| Suv420h1 | NM_001108512.1 |
| Slc38a2 | NM_181090.2 |
| Rgl1 | NM_001105957.1 |
| Lats2 | NM_001107267.1 |
| Ddc | NM_001270853.1 |
| Rbpms2 | NM_001173426.1 |
| Npc1 | NM_153624.2 |
| Cdk16 | NM_031077.1 |
| Tnpo1 | NM_001100692.1 |
| Lhfpl2 | NM_001106402.1 |
| Arhgef15 | NM_001105789.1 |
| Itga5 | NM_001108118.1 |
| Ugp2 | NM_001024743.1 |
| Bsdc1 | NM_001106636.1 |
| Isca1 | NM_181626.3 |
| Rimbp2 | NM_001100488.2 |
| Osbpl8 | NM_001309455.1 |
| Pcmtd1 | NM_001257345.1 |
| Klhl29 | NM_001106713.2 |
| Sort1 | NM_031767.1 |
| Grip2 | NM_138535.2 |
| Slc24a3 | NM_053505.2 |
| Insig1 | NM_022392.1 |
| Dyrk2 | NM_001108100.1 |
| Fkbp1a | NM_013102.3 |
| Dusp5 | NM_133578.1 |
| Nefh | NM_012607.2 |
| Slc25a32 | NM_001173334.1 |
| Pon2 | NM_001013082.1 |
| Peak1 | NM_001108149.1 |
| Nox4 | NM_053524.1 |
| Scaf11 | NM_001271170.1 |
| Btg2 | NM_017259.1 |
| Slc17a6 | NM_053427.1 |
| Ppp1r12c | NM_001191946.2 |
| Ube2z | NM_001037643.2 |
| Abhd13 | NM_001271072.1 |
| Klf4 | NM_053713.1 |
| Rsbn1 | NM_001191710.1 |
| Tmem229a | NM_001109480.1 |
| Aars | NM_001100517.1 |
| Spryd4 | NM_001037765.1 |
| Pip5k1c | NM_001033970.1 |
| Snap29 | NM_053810.3 |
| Bcl2l11 | NM_171988.2 |
| Cldn11 | NM_053457.2 |
| Usp28 | NM_001108144.1 |
| Prkar1b | NM_001033679.1 |
| Plekhb2 | NM_001106899.1 |
| Tef | NM_019194.2 |
| Ccnjl | NM_001037773.4 |
| Mier3 | NM_001168000.1 |
| Tgif1 | NM_001015020.1 |
| Slc12a5 | NM_134363.1 |
| Fam20c | NM_001012238.1 |
| Tcf21 | NM_001032397.1 |
| Synj1 | NM_053476.2 |
| Wasl | NM_001110365.1 |
| Gata2 | NM_033442.1 |
| Nlk | NM_001191924.1 |
| Vav3 | NM_001191714.1 |
| Pak7 | NM_001107781.1 |
| Ube2v1 | NM_001110345.2 |
| Snrk | NM_138833.1 |
| Rqcd1 | NM_001009357.1 |
| Med13 | NM_001107035.1 |
| Csnk2a2 | NM_001107409.1 |
| Ets1 | NM_012555.2 |
| Dram1 | NM_001173427.1 |
| Dmrta1 | NM_001107945.1 |
| Serinc1 | NM_182951.1 |
| Patl1 | NM_001108520.2 |
| Taf5 | NM_001106365.1 |
| Tbk1 | NM_001106786.1 |
| Errfi1 | NM_001014071.1 |
| Mapk8 | NM_053829.2 |
| Enc1 | NM_001003401.1 |
| Cmtr2 | NM_001106186.1 |
| Slc6a15 | NM_172321.1 |
| Tbl1xr1 | NM_001108941.1 |
| Nmnat1 | NM_001037556.2 |
| Mobp | NM_012720.1 |
| Bmper | NM_001135799.1 |
| Zfp644 | NM_001139484.1 |
| Inadl | NM_080398.1 |
| Tm2d2 | NM_001017444.1 |
| Rras2 | NM_001013434.1 |
| Nutf2 | NM_001007629.1 |
| Slc44a1 | NM_001033852.1 |
| Naa15 | NM_001107674.1 |
| Rock2 | NM_013022.2 |
| Fech | NM_001108434.1 |
| Art4 | NM_001173509.1 |
| Vwc2l | NM_001109308.1 |
| Ecel1 | NM_021776.1 |
| Ranbp10 | NM_001135875.1 |
| Ccdc64 | NM_001191667.1 |
| Slc24a2 | NM_031743.2 |
| Cnot7 | NM_001107313.1 |
| Ppp1r12b | NM_001107178.3 |
| LOC100125367 | NM_001103357.1 |
| Lrp6 | NM_001107892.1 |
| Ccdc34 | NM_001108587.1 |
| Gtpbp2 | NM_001013225.2 |
| Ppp4r2 | NM_001106613.2 |
| Kitlg | NM_021843.4 |
| Hipk1 | NM_001100986.1 |
| Arl1 | NM_022385.3 |
| Gad1 | NM_017007.1 |
| Cyp2s1 | NM_001107495.1 |
| Psme3 | NM_001011894.1 |
| Cipc | NM_001108044.2 |
| Per2 | NM_031678.1 |
| Atp11a | NM_001107324.2 |
| Ppm1b | NM_033096.2 |
| Adipor1 | NM_207587.1 |
| Zfp36 | NM_133290.3 |
| Tmeff2 | NM_001108795.1 |
| Lpar4 | NM_001106940.1 |
| Cpt1a | NM_031559.2 |
| Hmgcs1 | NM_017268.1 |
| Gria2 | NM_001083811.1 |
| Pbx3 | NM_001107834.1 |
| Mapk6 | NM_031622.2 |
| Raph1 | NM_001108798.2 |
| Cks2 | NM_001126083.1 |
| RGD1565616 | NM_001109206.1 |
| Prpf4b | NM_001011923.1 |
| Elmod2 | NM_001109506.1 |
| RGD1310553 | NM_001008517.1 |
| Hspbp1 | NM_139261.1 |
| Szrd1 | NM_001114599.2 |
| Anks6 | NM_001015028.2 |
| RGD1310127 | NM_001035517.1 |
| Aldh1a2 | NM_053896.2 |
| Lrrc41 | NM_001009710.1 |
| Irak1 | NM_001127555.1 |
| Sacm1l | NM_053798.2 |
| Shisa7 | NM_001145175.1 |
| Necab3 | NM_001098724.1 |
| Gypc | NM_001013233.1 |
| P2rx7 | NM_019256.1 |
| Stam | NM_001109121.2 |
| Cenpj | NM_001107265.1 |
| Snx11 | NM_001012012.2 |
| Acot2 | NM_138907.2 |
| Tmem216 | NM_001271039.1 |
| Cox18 | NM_001106000.1 |
| Cmc1 | NM_001199225.1 |
| Ccdc25 | NM_001108382.1 |
| Mrrf | NM_001008354.1 |
| Mmd | NM_001007673.1 |
| Ywhaz | NM_013011.3 |
| Cdk17 | NM_001108082.2 |
| Zfp202 | NM_001109290.1 |
| Psmf1 | NM_001101005.1 |
| Lrp4 | NM_031322.3 |
| Pacs1 | NM_134406.1 |
| Zbtb39 | NM_001130537.1 |
| Tspan18 | NM_001107750.1 |
| Drgx | NM_145767.1 |
| Cry2 | NM_133405.2 |
| Grem2 | NM_001105974.1 |
| Ctsw | NM_001024242.1 |
| Dda1 | NM_001134790.1 |
| Srsf3 | NM_001047907.3 |
| Paqr5 | NM_001014092.1 |
| Fbxw8 | NM_001107145.1 |
| Dag1 | NM_053697.1 |
| Pla2g2f | NM_001109587.1 |
| Cd6 | NM_175577.3 |
| Tnpo2 | NM_001107166.1 |
| Prkx | NM_001033963.1 |
| Mllt1 | NM_001106876.1 |
| Lipa | NM_012732.3 |
| Cep85 | NM_001080151.1 |
| Gpr155 | NM_001107811.1 |
| Sox10 | NM_019193.2 |
| Cdc25a | NM_133571.1 |
| Slc23a2 | NM_017316.2 |
| Utp11l | NM_001107978.2 |
| Rpgrip1l | NM_001107414.1 |
| Actr3 | NM_031068.1 |
