# Supplementary material for: Sinomenine attenuates uremia vascular calcification by miR-143-5p
Source: Sci Rep. 2025 Jan 13;15:1798. doi: 10.1038/s41598-025-86055-2 (PMC11730593; doi:10.1038/s41598-025-86055-2)
Supplement: Supplementary file 15 — Supplementary Material 15 [file 41598_2025_86055_MOESM15_ESM.docx]

**Supplementary Figure Legends**

**Supplementary Figure S1 Laboratory parameters of animal model.**

1. Blood urea nitrogen. (B) Blood creatinine. (C) Blood calcium. (D) Blood phosphate. Error bars indicate standard deviations. One-way analysis of variance was used. **P* < 0.05, ***P* < 0.01, ****P* < 0.001. NS, not significant. Con, control group; CKD, chronic kidney disease group; SIN 20mg, sinomenine 20 mg/kg/day group; SIN 40mg, sinomenine 40 mg/kg/day.

**Supplementary Figure S2 Sinomenine alleviated vascular calcification in adenine-induced uremic rats in different doses.**

1. Micro-CT images of aortas in Con, CKD, SIN 20mg, SIN 40mg. (B) Micro-CT images of partial aorta from three corresponding group showed calcified lesions in rectangle area of (A). (C) Calcification depositions were quantified and normalized to μg Ca/mg protein weight. n = 3 per group. Error bars indicate standard deviations. One-way analysis of variance was used. **P* < 0.05, ***P* < 0.01. NS, not significant. Con, control group; CKD, chronic kidney disease group; SIN 20mg, sinomenine 20 mg/kg/day group; SIN 40mg, sinomenine 40 mg/kg/day.

**Supplementary Figure S3 MiRNA-seq uncovered** **differentially expressed miRNAs in uremic rat aortas (Log 2 |FC| >1.0, *q* < 0.05).**

(A) The volcano plots of differentially expressed miRNAs between Con and CKD. (B) The volcano plots of differentially expressed miRNAs between Sin and CKD. (C) The heatmap of 11 differentially expressed miRNAs between Con and CKD. (D) The heatmap of 5 differentially expressed miRNAs between Sin and CKD. n = 3 per group. Log 2 |FC| > 1.0, *q* < 0.05. Con, control group; CKD, chronic kidney disease group; Sin, sinomenine 40 mg/kg/d group.

**Supplementary Figure S4 Gene Ontology analyses for target genes of 9 differentially expressed miRNAs (Log 2 |FC| > 0.585, *p* < 0.05).**

1. GO enrichment for target genes of rno-miR-143-5p. (B) GO enrichment for target genes of 8 screened differentially expressed miRNAs. GO (Gene Ontology).

**Supplementary Figure S5 Kyoto Encyclopedia of Genes and Genomes analyses for target genes of 9 differentially expressed miRNAs (Log 2 |FC| > 0.585, *p* < 0.05).**

1. KEGG analyses for target genes of rrno-miR-143-5p. (B) KEGG analyses for target genes of 8 differentially expressed miRNAs. KEGG, Kyoto Encyclopedia of Genes and Genomes.

**Supplementary Figure S6 Gene Ontology analyses for target genes of 16 differentially expressed miRNAs (Log 2 |FC| > 1.0, *q* < 0.05).**

(A) GO enrichment for target genes of 11 differentially expressed miRNAs between Con and CKD. (B) GO enrichment for target genes of 5 differentially expressed miRNAs between Sin and CKD. GO (Gene Ontology); Con, control group; CKD, chronic kidney disease group; Sin, sinomenine 40 mg/kg/d group.

**Supplementary Figure S7 Kyoto Encyclopedia of Genes and Genomes analyses for target genes of 16 differentially expressed miRNAs (Log 2 |FC| > 1.0, *q* < 0.05).**

1. KEGG analyses for target genes of 11 differentially expressed miRNAs between Con and CKD. (B) KEGG analyses for target genes of 5 differentially expressed miRNAs between Sin and CKD. KEGG, Kyoto Encyclopedia of Genes and Genomes; Con, control group; CKD, chronic kidney disease group; Sin, sinomenine 40 mg/kg/d group.

**Supplementary Figure S8 Validation of 8 screened miRNAs by qRT-PCR.**

Relative expression of 8 miRNAs in aortas tissue in Con, CKD and Sin. (A) rno-miR-208a-3p. n = 6 per group. (B) rno-miR-219a-2-3p. n = 6 per group. (C) rno-miR-223-5p. n = 6 per group. (D) rno-miR-323-3p. n = 6 per group. (E) rno-miR-409b. n = 6 per group. (F) rno-miR-433-3p. n = 4 per group. (G) rno-miR-485-5p. n = 6 per group. (H) rno-miR-92b-3p. n = 6 per group. Error bars indicate standard deviations. One-way analysis of variance was used. NS, not significant.

**Supplementary Figure S9 Different concentrations of sinomenine protective effect on A7R5 cells calcification in high phosphate condition.**

Representative Alizarin Red S staining images showed sinomenine reduced calcification crystallization in high phosphate condition both macroscopically and microscopically in different concentrations. NC, negative control; LP, low phosphate; HP, high phosphate; SIN 20, 20 μg/mL sinomenine; SIN 60, 60 μg/mL sinomenine; SIN 100, 100 μg/mL sinomenine. Scale bar: 1mm.

**Supplementary Figure S10 Cell counting kit-8 of different concentrations of sinomenine at 24 h.**

Sinomenine didn’t indicate MOVAS-1 cells inhibition at 24 h. n = 3 per group. Error bars indicate standard deviations. One-way analysis of variance was used. NS, not significant.

**Supplementary Figure S11 Calcification crystallization images in higher magnification power of microscope of Figure 7G.**

Representative alizarin red staining showed mmu-miR-143-5p mimic decreased calcification crystallization compared with NC mimic in high phosphate condition on seventh day, while mmu-miR-143-5p inhibitor increased calcification crystallization compared with NC inhibitor in high phosphate condition. NC, negative control; HP, high phosphate. Scale bar: 400 μm.

**Supplementary Figure S12 Calcification crystallization images in higher magnification power of microscope of Figure 7H.**

Representative alizarin red staining showed sinomenine decreased calcification crystallization in NC mimic and NC inhibitor compared with HP. Moreover, mmu-miR-143-5p mimic showed a stronger calcification crystallization decrease compared with NC mimic or mmu-miR-143-5p inhibitor high phosphate condition. NC, negative control; HP, high phosphate; SIN, 40 μg/mL sinomenine. Scale bar: 400 μm.

**Supplementary Figure S13 Mixed analyses of mmu-miR-143-5p and sinomenine on vascular calcification in MOVAS-1 cell.**

Calcification depositions were quantified and normalized to μg Ca/mg protein weight. (A) Sinomenine decreased calcification deposition with or without miRNA mimic transfection. (B) mmu-miR-143-5p mimic decreased calcification crystallization in high phosphate condition compared with NC mimic. NC, negative control; HP, high phosphate; SIN, 40 μg/mL sinomenine.

(C) Sinomenine decreased calcification deposition with or without miRNA inhibitor transfection. (D) mmu-miR-143-5p inhibitor increased calcification crystallization in high phosphate condition compared with NC inhibitor. The difference was not significant between mmu-miR-143-5p inhibitor and NC inhibitor in high phosphate condition with sinomenine. NC, negative control; HP, high phosphate; SIN, 40 μg/mL sinomenine.
